# HSPB8 frameshift mutant aggregates weaken chaperone-assisted selective autophagy in neuromyopathies

**DOI:** 10.1080/15548627.2023.2179780

**Published:** 2023-02-28

**Authors:** Barbara Tedesco, Leen Vendredy, Elias Adriaenssens, Marta Cozzi, Bob Asselbergh, Valeria Crippa, Riccardo Cristofani, Paola Rusmini, Veronica Ferrari, Elena Casarotto, Marta Chierichetti, Francesco Mina, Paola Pramaggiore, Mariarita Galbiati, Margherita Piccolella, Jonathan Baets, Femke Baeke, Riet De Rycke, Vincent Mouly, Tommaso Laurenzi, Ivano Eberini, Anna Vihola, Bjarne Udd, Lan Weiss, Virginia Kimonis, Vincent Timmerman, Angelo Poletti

**Affiliations:** a Dipartimento di Scienze Farmacologiche e Biomolecolari “Rodolfo Paoletti”, Dipartimento di Eccellenza 2018-2027, Università degli Studi di Milano, Milan, Italy; b Unit of Medical Genetics and Neurogenetics, Fondazione IRCCS Istituto Neurologico Carlo Besta, Milan, Italy; c Peripheral Neuropathy Research Group, Department of Biomedical Sciences and Institute Born Bunge, University of Antwerp, Antwerpen, Belgium; d Neuromics Support Facility, VIB Center for Molecular Neurology, VIB, Antwerp, Belgium; e Neuromics Support Facility, Department of Biomedical Sciences, University of Antwerp, Antwerp, Belgium; f Laboratory of Neuromuscular Pathology, Institute Born Bunge, and Translational Neurosciences, Faculty of Medicine and Health Sciences, University of Antwerp, Antwerp, Belgium; g Neuromuscular Reference Centre, Department of Neurology, Antwerp University Hospital, Antwerp, Belgium; h Department of Biomedical Molecular Biology, and VIB Center for Inflammation Research, and VIB Bioimaging Core, Ghent University Ghent, Belgium; i Sorbonne Université, Inserm, Institut de Myologie, Centre de Recherche en Myologie, Paris, France; j Folkhälsan Research Center, University of Helsinki, Helsinki, Finland; k Neuromuscular Research Center, Tampere University Hospital, Tampere, Finland; l Vasa Central Hospital, Vasa, Finland; m Department of Pediatrics, University of California, Irvine, Lombardy, United States

**Keywords:** BAG3, CASA, HSPA, HSPB8, misfolding, myopathy, neuromuscular disorders, neuropathy, protein quality control

## Abstract

Chaperone-assisted selective autophagy (CASA) is a highly selective pathway for the disposal of misfolding and aggregating proteins. In muscle, CASA assures muscle integrity by favoring the turnover of structural components damaged by mechanical strain. In neurons, CASA promotes the removal of aggregating substrates. A crucial player of CASA is HSPB8 (heat shock protein family B (small) member 8), which acts in a complex with HSPA, their cochaperone BAG3, and the E3 ubiquitin ligase STUB1. Recently, four novel *HSPB8* frameshift (fs) gene mutations have been linked to neuromyopathies, and encode carboxy-terminally mutated HSPB8, sharing a common C-terminal extension. Here, we analyzed the biochemical and functional alterations associated with the HSPB8_fs mutant proteins. We demonstrated that HSPB8_fs mutants are highly insoluble and tend to form proteinaceous aggregates in the cytoplasm. Notably, all HSPB8 frameshift mutants retain their ability to interact with CASA members but sequester them into the HSPB8-positive aggregates together with two autophagy receptors SQSTM1/p62 and TAX1BP1. This copartitioning process negatively affects the CASA capability to remove its clients and causes a general failure in proteostasis response. Further analyses revealed that the aggregation of the HSPB8_fs mutants occurs independently of the other CASA members or from the autophagy receptors interaction, but it is an intrinsic feature of the mutated amino acid sequence. HSPB8_fs mutants aggregation alters the differentiation capacity of muscle cells and impairs sarcomere organization. Collectively, these results shed light on a potential pathogenic mechanism shared by the HSPB8_fs mutants described in neuromuscular diseases.

**Abbreviations** : ACD: α-crystallin domain; ACTN: actinin alpha; BAG3: BAG cochaperone 3; C: carboxy; CASA: chaperone-assisted selective autophagy; CE: carboxy-terminal extension; CLEM: correlative light and electron microscopy; CMT2L: Charcot-Marie-Tooth type 2L; CTR: carboxy-terminal region; dHMNII: distal hereditary motor neuropathy type II; EV: empty vector; FRA: filter retardation assay; fs: frameshift; HSPA/HSP70: heat shock protein family A (Hsp70); HSPB1/Hsp27: heat shock protein family B (small) member 1; HSPB8/Hsp22: heat shock protein family B (small) member 8; HTT: huntingtin; KO: knockout; MAP1LC3B/LC3: microtubule associated protein 1 light chain 3 beta; MD: molecular dynamics; MTOC: microtubule organizing center; MYH: myosin heavy chain; MYOG: myogenin; NBR1: NBR1 autophagy cargo receptor; CALCOCO2/NDP52: calcium binding and coiled-coil domain 2; NSC34: Neuroblastoma X Spinal Cord 34; OPTN: optineurin; polyQ: polyglutamine; SQSTM1/p62: sequestosome 1; STUB1/CHIP: STIP1 homology and U-box containing protein 1; TARDBP/TDP-43: TAR DNA binding protein; TAX1BP1: Tax1 binding protein 1; TUBA: tubulin alpha; WT: wild-type.

## Introduction

Small heat shock proteins are ATP-independent molecular chaperones that act as first responders to protein aggregation. The HSPB protein family is composed of ten members, many of which are ubiquitously expressed, and which fulfill diverse functions under basal and stress conditions. HSPB protein family members share a conserved α-crystallin domain (ACD), that is flanked by intrinsically disordered sequences on either side [1]. Although the N- and C- terminal regions (NTR, CTR) are variable, short sequences are common in some members of the HSPB family, such as the SRLFDQxFG and the IxI/V motifs in the NTR and CTR, respectively [2,3]. HSPBs recognize misfolding proteins in a near-native state, preventing their further misfolding and facilitating the refolding by ATP-dependent heat shock proteins like the HSPA/HSP70 (heat shock protein family A (Hsp70)) [4].

HSPB8 (heat shock protein family B (small) member 8) is a member of the HSPB family and forms a stable protein complex with the chaperone HSPA, the cochaperone BAG3 (BAG3 cochaperone 3), and the E3-ubiquitin ligase STUB1/CHIP (STIP1 homology and U-Box containing protein 1) [5–7]. The complex is better known as the chaperone-assisted selective autophagy (CASA) complex and acts in protein refolding and degradation (recently reviewed in [8]). Damaged and misfolded substrates recognized by the HSPB8 and HSPA chaperones are subjected to HSPA-driven refolding or to STUB1-mediated ubiquitination [5,9]. Ubiquitinated substrates are degraded through the autophagosome-lysosomal pathway, by recruiting selective autophagy receptors, like SQSTM1/p62 (sequestosome 1), which bridge them to the lipidated MAP1LC3B/LC3 (microtubule associated protein 1 light chain 3 beta) exposed on phagophore membranes [10]. A failure in the degradation of misfolded substrates may result in their compartmentalization into cytoplasmic deposits, such as aggresomes [11,12]. Indeed, by interacting with the dynein motor complex, BAG3 can re-route misfolded substrates to the microtubule organizing center (MTOC) to aggresomes. HSPB8, together with BAG3 and HSPA, has been also involved in stress granules maintenance, or granulostasis [13]. In both CASA and granulostasis, HSPB8 can intervene upstream to BAG3 and HSPA, by recognizing defective substrates and exerting its holdase activity [13,14].

The CASA complex has been mainly studied in the skeletal muscle and neuronal tissues. In muscles, proteins that form Z-disks are subjected to continuous mechanical stress, leading to the recruitment of the CASA complex, which prevents protein misfolding and facilitates the degradation of damaged structural proteins [5,15]. In neurons, the CASA complex promotes the removal of a broad variety of misfolded substrates causative of neurodegenerative diseases [6,16–20]. Mutations in CASA complex components have been associated with different neuromuscular conditions: BAG3 mutations are associated with myopathies, neuropathies, and also cardiomyopathies [21–28]; STUB1 mutations are linked to spinocerebellar ataxias [29–32]; HSPB8 mutations cause distal hereditary motor neuropathy type II (dHMNII), Charcot-Marie-Tooth type 2 L (CMT2L) disease, and myopathies [33–36].

The most common and characterized HSPB8 mutations are the dominantly inherited missense mutations targeting the conserved lysine 141 residue (K141E/N/T/M), while the dominant HSPB8 N138T and the de novo P90L mutations have been reported once [34–36]. HSPB8 K141 mutations have been mainly described in motor neuropathies (as HSPB8 P90L and N138T), with few reports that associated this substitution (HSPB8 K141E) with rimmed vacuolar myofibrillar myopathy [33,37]. Instead, rimmed vacuolar myofibrillar myopathy is the predominant clinical phenotype of a group of dominantly inherited or de novo frameshift (fs) mutations in HSPB8 (HSPB8_fs) recently reported in different independent studies: the mutations p.P173Sfs*43 (c.515dupC), p.Q170Gfs*45 (c.508–509delCA), p.T194Sfs*23 (c.577–580dupGTCA), and p.T176Wfs*38 (c.525–529delAACAT) [38–41]. Only the HSPB8 p.P173Sfs*43 was also reported in myopathy with neurological involvement [33]. All these HSPB8_fs mutations fall in the carboxy (C) -terminus of HSPB8 and result in the production of a novel C-terminal sequence, which is longer than the original protein sequence. It has been suggested that HSPB8_fs mutations cause HSPB8 haploinsufficiency [38,40]. Alternatively, the HSPB8_fs mutations have been proposed to alter the CASA pathway, triggering insufficient disposal of damaged and misfolded substrates. This second hypothesis is based on the observation that the autophagic markers LC3 and SQSTM1 accumulate in patient fibroblasts and in vacuolated fibers of a patient’s muscle biopsy [33,38,42].

To unravel the molecular mechanisms underlying the HSPB8_fs mutations pathology, we studied the molecular behavior of three HSPB8_fs mutants and found that the HSPB8_fs mutants cause a toxic gain-of-function. Our data demonstrated dysregulation of the HSPB8_fs mutants, resulting in their accumulation into cytoplasmic aggregates. Since HSPB8_fs mutants maintained their ability to dimerize and interact with BAG3, they recruited and sequestered the CASA complex components together with the autophagy receptors SQSTM1 and TAX1BP1 (Tax1 binding protein 1), which all accumulated into cytoplasmic aggregates. Moreover, we revealed an essential role for BAG3 in positioning chaperone-substrate complexes, and we showed that the HSPB8_fs mutants induced a proteostasis impairment, supported by an increase in insoluble ubiquitinated substrates, and interfered with CASA function. Analyses of the modified and elongated C-terminus of HSPB8_fs mutants suggested that this amino acid tract is intrinsically disordered, increasing the HSPB8_fs tendency to accumulate in cytoplasmic aggregates. Altogether, our data revealed novel insights into how C-terminal fs mutations weaken the CASA complex functionality and impair cell proteostasis representing a common cause for HSPB8-associated neuromuscular phenotypes.

## Results

### The HSPB8_fs mutants share a common elongated C-terminal tail causing HSPB8 insolubility and aggregation

**Figure 1.**
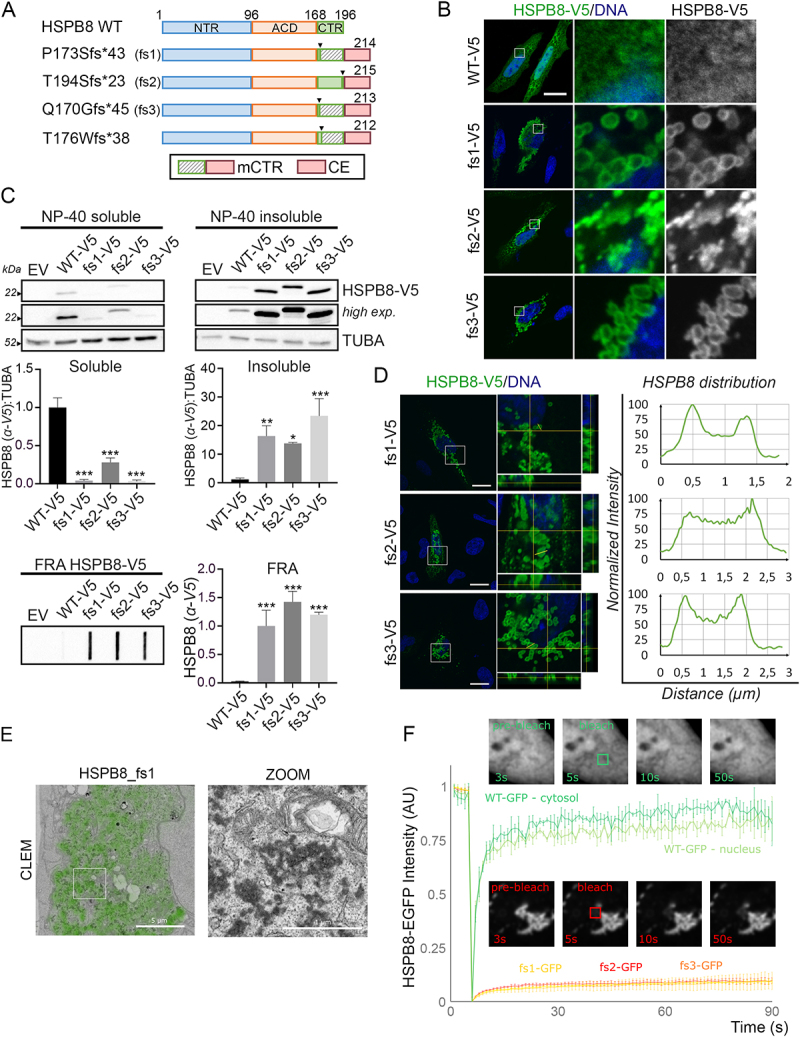
Frameshift mutants of HSPB8 form high molecular weight insoluble species and cytoplasmic aggregates. (A) Schematic representation of HSPB8_WT and its HSPB8_fs mutant structures. The α-crystallin domain (ACD) is reported in Orange, the N-terminal region (NTR) in blue, the C-terminal region (CTR) in green. The striped green region represents the mutated CTR of p.P173Sfs*43 (fs1), p.Q170Gfs*45 (fs3) and p.T176Wfs*38 mutants. The red region represents the common C-terminal extension (CE) shared by all fs mutants. The box at the bottom reports the nomenclature of the mutated C-termini of the HSPB8 mutants: mCTR (mutated CTR, comprises both the mutated CTR and the CE) and the CE. (B) Immunofluorescence analysis of HeLa cells transiently transfected with V5-tagged HSPB8 constructs. HSPB8 is in green, nuclei were stained with DAPI, scale bar: 20 μm. (C) Western blot and filter retardation assay (FRA) analyses of NP-40 soluble/insoluble protein fractions of HeLa cells transiently transfected with V5-tagged HSPB8 constructs or an empty vector (EV). Bar graphs report mean values (± SD) of densitometry of HSPB8-V5 on soluble TUBA/tubulin alpha for western blot. All graphs are normalized to the HSPB8_WT-V5. One-way ANOVA with Tukey’s test was performed: * p < 0.05, ** p < 0.01, *** p < 0.001; n = 3. (D) Distribution of HSPB8_fs mutants in the hollow aggregates they formed in HeLa cells transiently transfected with V5-tagged HSPB8 constructs. To visualize the confocal volume, orthogonal XZ and YZ projections at the yellow crosshairs of the XY image are shown. The plots report the normalized fluorescence intensities of the V5-tagged HSPB8 signals (y axis) with respect to the distance (µm; x axis) of the yellow segment. HSPB8 (α-V5) is in green, nuclei were stained with DAPI, scale bar: 20 μm. (E) Correlative light and electron microscopy (CLEM) on HeLa cells transiently transfected with a representative HSPB8_fs mutant (fs1). The HSPB8-GFP signal imaged on the confocal microscope (green) overlaid onto a low magnification transmission electron microscopy image is shown on the left. Details of the (dark) protein dense aggregates can be observed via the boxed zoom on the right. CLEM Scale bar: 5 μm; zoom: 1 µm. (F) Fluorescence recovery after photobleaching (FRAP) analysis on HeLa cells transiently transfected with GFP-tagged HSPB8 constructs and imaged every second. After 5 s, a square region (2.8 µm x 2.8 µm) was bleached, and the recovery was monitored over the next 85s. Example images at indicated times are shown of HSPB8_wt-GFP (green) and HSPB8_fs2-GFP (red). Graph bar indicates the means ± SD of fluorescence intensities over time (n = 3). The constructs were abbreviated as follows: empty vector (EV), V5-tagged HSPB8_WT (WT-V5), V5-tagged HSPB8_fs mutants (fs1-V5, fs2-V5, fs3-V5), GFP-tagged HSPB8 mutants (WT-GFP, fs1-GFP, fs2-GFP, fs3-GFP).

The novel HSPB8_fs mutations associated with neuromuscular diseases are predicted to encode for elongated HSPB8 proteins. Analysis of the new C-terminal sequence, derived from fs mutations at various positions in the C-terminus, revealed that different mutants result in a very similar modification of the CTR of HSPB8, as for the p.P173Sfs*43, p.Q170Gfs*45 and p.T176Wfs*38 mutants, and the addition of an identical carboxy-terminal extension (CE) in all mutated HSPB8s (Figure 1A and **S1A;** Table 1). To gain insight into the molecular mechanisms underlying disease-associated HSPB8_fs mutations, we overexpressed V5-tagged wild-type (WT-V5) or mutated HSPB8s (p.P173Sfs*43 [fs1-V5], p.T194Sfs*23 [fs2-V5], and p.Q170Gfs*45 [fs3-V5]). Subcellular localization analysis revealed an altered distribution of all three HSPB8_fs mutants with respect to the HSPB8_WT in HeLa cells (Figure 1B). While HSPB8_WT correctly diffused in the cytoplasm, the HSPB8_fs mutants accumulated into cytoplasmic structures, gathering at the perinuclear region. Importantly, this altered distribution was also observed in HSPB8-V5-transduced human myoblasts, and in Neuroblastoma X Spinal Cord 34 (NSC34) cells transfected with untagged HSPB8 constructs (**Figure S1B**), the latter confirming that the tendency of the HSPB8_fs mutants to deposit in the cytoplasm is not dependent on the V5-tag at the C-terminus. Accordingly, we observed that all HSPB8_fs mutants were detectable by western blot and displayed a preferential partitioning in the NP-40 insoluble fractions, in which their levels were higher than the HSPB8_WT. In addition, HSPB8_fs mutants formed high molecular weight insoluble species, as shown in the filter retardation assay (FRA) (Figure 1C). Closer inspection of the cytoplasmic assemblies revealed that the two HSPB8_fs mutants with the altered CTR (fs1 and fs3) formed regular round-shaped structures with a hollow core exceeding 1 µm in diameter, resembling large vesicular structures. Conversely, the HSPB8_fs2 assemblies were more irregularly shaped and resembled typical aggregate-like structures (Figure 1D and **S1B**).

**Table 1.** HSPB8 frameshift genetic variants and features of the C-terminus.

Annotation	Variant	Mutation	Protein product (AA)	Differential C-terminus?	C-terminal elongation?
WT	-	-	196	-	-
Fs1	c.515dupC	p.P173Sfs*43	215	Yes	Yes
Fs2	c.577_580dupGTCA	p.T194Sfs*23	216	No	Yes
Fs3	c.508_509delCA	p.Q170Gfs*45	214	Yes	Yes
-	c.525_529delAACAT	p.T176Wfs*38	213	Yes	Yes

Given the appearance of vesicle-like structures, we wondered whether the HSPB8 signal for fs1 and fs3 mutants could be associated with membrane-enclosed organelles. We therefore tested for colocalization with mitochondria, endoplasmic reticulum, lipid droplets, autophagosomes, lysosomes, and stress granules as assessed by confocal microscopy using organelle-specific markers. However, the HSPB8_fs1, used as a representative mutant, displayed no association with any of these organelles (**Figure S1C**). Next, we used electron microscopy to resolve the ultrastructural details of the structures formed by the HSPB8_fs mutants. A correlative light and electron microscopy (CLEM) workflow was used to identify the sites of GFP-tagged HSPB8_fs mutant accumulations on the electron microscope (Figure 1E). Dense cytoplasmic amorphous globular-like protein aggregates formed by the HSPB8_fs1 were observed in the cytoplasm, which were not associated with any organelles or membranes. Comparable structures were observed in cells expressing the HSPB8_fs2 and fs3 (**Figure S1D**). To better define the dynamic properties of the HSPB8_fs mutant structures, we performed a fluorescence recovery after photobleaching (FRAP) assay on HeLa cells transfected with GFP-tagged HSPB8 constructs. Photobleaching of HSPB8-GFP WT in the nucleus or cytoplasm resulted in a fast recovery, with mobile fractions exceeding 75%, consistent with soluble protein. In contrast, recovery of aggregated HSPB8_fs was much slower with mobile FRAP fractions below 10%, indicating that the immobile aggregates consist of insoluble proteins, without significant HSPB8 subunit exchange (Figure 1F). Collectively, these results demonstrate that HSPB8_fs mutations encode protein products characterized by increased insolubility and cytoplasmic aggregation in overexpressing cells.

### HSPB8_fs mutants interact with HSPB8_WT and CASA members causing their sequestration

**Figure 2.**
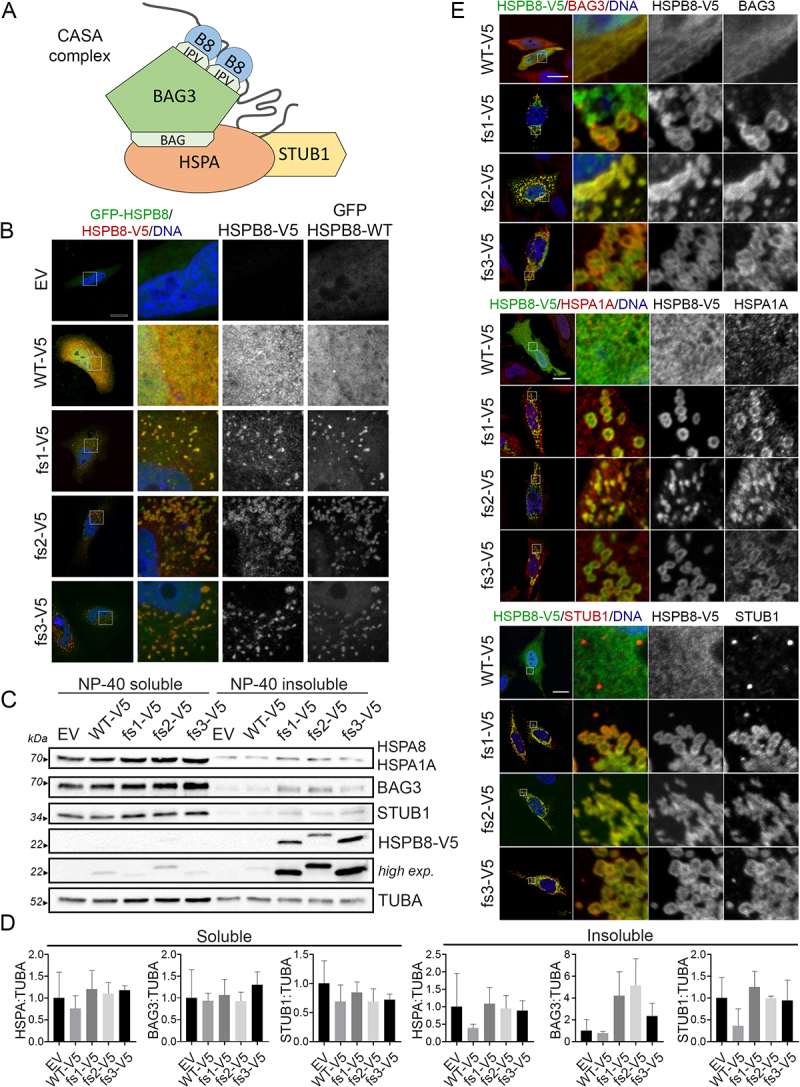
The HSPB8_fs mutants interact with CASA members causing their sequestration. (A) Schematic representation of the CASA complex main members: the co-chaperone BAG3 interacts with HSPA through the BAG3 domain, and with HSPB8 (indicated as B8) through the IPV motifs. STUB1 takes interaction with HSPA. The gray line represents a CASA client recognized by the chaperones. (B) Immunofluorescence analysis of HeLa cells transiently co-transfected with V5-tagged HSPB8 constructs (red) and GFP-tagged HSPB8 WT. Nuclei were stained with DAPI, scale bar: 20 μm. (C) Western blot analysis of NP-40 soluble/insoluble protein fractions of HeLa cells transiently transfected with V5-tagged HSPB8 constructs or an empty vector. (D) Bar graphs report mean values (± SD) of densitometry of BAG3, HSPA (W27 clone), STUB1 on soluble TUBA. All graphs are normalized to control cells (EV). One-way ANOVA with Tukey’s test was performed; n = 3. (E) Immunofluorescence analyses of HeLa cells transiently transfected with V5-tagged HSPB8 constructs (green) and stained for BAG3 (Top panel, red), or HSPA1A (Middle panel, red), or STUB1 (Bottom panel, red). Nuclei were stained with DAPI, scale bar: 20 μm. The constructs were abbreviated as follows: empty vector (EV), V5-tagged HSPB8_WT (WT-V5), V5-tagged HSPB8_fs mutants (fs1-V5, fs2-V5, fs3-V5).

HSPB8 forms homodimers and interacts with BAG3 in a 2:1 stoichiometry [43,44]. This may initiate CASA complex assembly since BAG3 additionally recruits HSPA and STUB1 (Figure 2A). We first investigated the effect of the HSPB8_fs mutants on the HSPB8_WT protein. HeLa cells were transiently co-transfected with V5-tagged HSPB8 constructs and a GFP-tagged HSPB8_WT (Figure 2B). We observed a diffuse distribution of the GFP-tagged HSPB8_WT protein in cells co-transfected with an empty vector or V5-tagged HSPB8_WT. Conversely, the V5-tagged HSPB8_fs mutants were able to sequester the GFP-tagged HSPB8_WT protein, suggesting a dominant negative effect of the mutants on the WT protein.

To confirm this observation, we took advantage of a HEK293T cell line stably overexpressing a V5-tagged HSPB8_WT [45]. Transient transfection of untagged HSPB8_fs constructs in HEK293T-HSPB8-WT-V5 resulted in increased protein levels and high molecular weight insoluble species of the V5-tagged HSPB8_WT detected by FRA analysis, supporting that the HSPB8_WT is entrapped in HSPB8_fs mutant aggregates (**Figure S2A**). Using the same cell model, we evaluated the ability of the HSPB8_fs mutants to dimerize with the HSPB8_WT and to interact with BAG3. To this purpose, we analyzed transfected HEK293T-HSPB8-WT-V5 RIPA-soluble protein extracts by co-immunoprecipitation analysis using an anti-V5 tag or an anti-BAG3 antibody. We observed that both the untagged HSPB8_fs mutants and WT co-immunoprecipitated together with the V5-tagged HSPB8_WT and the BAG3 partner (**Figure S2B**). Since the RIPA-insoluble fractions containing the HSPB8_fs aggregates were removed from the analyses, these results suggest that HSPB8_fs mutants, in their soluble form, still interact with HSPB8_WT and BAG3, and that the other CASA members may be sequestered by HSPB8_fs mutants as a consequence of the specific interaction between HSPB8 and BAG3 proteins. Therefore, we performed an NP-40 soluble/insoluble protein extraction of HeLa cells transiently overexpressing V5-tagged HSPB8 proteins. As shown in Figure 2C,D, in the soluble fractions, HSPA, BAG3, and STUB1 protein levels were almost unaffected by HSPB8_WT or HSPB8_fs mutants. Instead, in the insoluble fractions, we observed a trend toward an increase in HSPA, BAG3, and STUB1 levels in HeLa cells expressing HSPB8_fs mutants compared to HeLa cells expressing HSPB8_WT. To assess whether the CASA complex members were recruited to HSPB8_fs aggregates, we evaluated the intracellular distribution of the different CASA complex members by immunofluorescence analysis. HeLa cells expressing the HSPB8_WT protein displayed a diffuse distribution of BAG3 and inducible HSPA1A, and a punctate pattern for STUB1 (Figure 2E). In contrast, BAG3, HSPA1A, and STUB1 co-partitioned with HSPB8_fs mutant proteins to form cytoplasmic aggregate structures.

Collectively, these data show that HSPB8_fs mutants may act in a dominant negative fashion on the HSPB8_WT by sequestering it, and dysregulate the distribution of the CASA members, possibly affecting the function of the CASA complex.

### HSPB8_fs mutants associate with ubiquitinated protein accumulations and defects in processing CASA substrates

**Figure 3.**
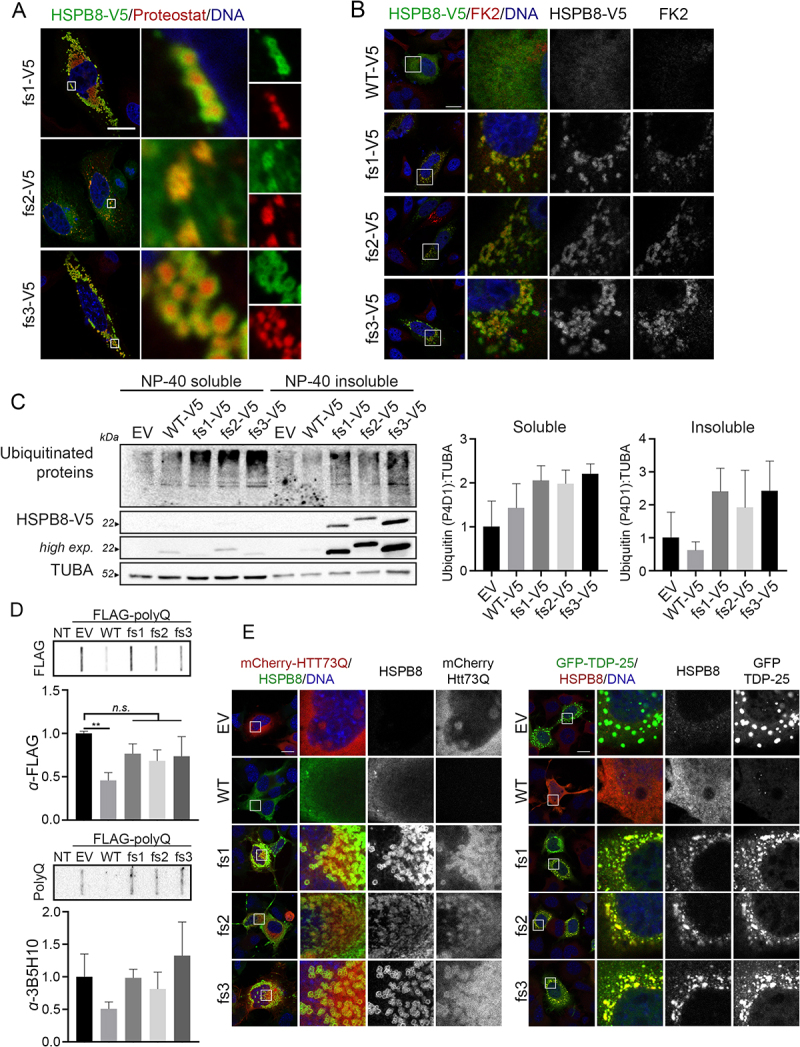
HSPB8_fs mutants associated with ubiquitinated protein accumulations and defects in processing CASA substrates. (A) Immunofluorescence analysis of HeLa cells transiently transfected with V5-tagged HSPB8_fs mutants constructs (green) and stained with Proteostat dye (red). Nuclei were stained with DAPI, scale bar: 20 μm. (B) Immunofluorescence analysis of HeLa cells transiently transfected with V5-tagged HSPB8 constructs (green) and stained for mono- and poly-ubiquitinated substrates (FK2, red). Nuclei were stained with DAPI, scale bar: 10 μm. (C) Western blot analysis of NP-40 soluble/insoluble protein extracts of HeLa transiently transfected with an empty vector or V5-tagged HSPB8 constructs. Bar graphs report mean values (± SD) of densitometry of ubiquitinated proteins on soluble TUBA. All graphs are normalized to control cells (EV). One-way ANOVA with Tukey’s test was performed; n = 3. (D) Filter retardation assay analyses on NSC34 transiently expressing a FLAG-tagged polyQ (74Q) construct and an empty vector or untagged HSPB8 constructs and analyzed for polyQ aggregation. Bar graphs report mean values (± SD) of densitometry of FLAG-polyQ on FRA. All graphs are normalized to the control (EV). One-way ANOVA with Dunnett’s test was performed, by comparing HSPB8s expressing cells to EV: ** p < 0.01; n = 3. (E) Immunofluorescence analyses on NSC34 transiently expressing an empty vector or untagged HSPB8 constructs with mCherry-HTT73Q (left, red) or GFP-TDP-25 (right, green). Staining was performed using an anti-HSPB8 antibody and nuclei were stained with DAPI, scale bar: 20 μm.

Given the ability of the HSPB8_fs mutants to sequester the other members of the CASA complex, we speculated that they may induce a defect in the handling and degradation of misfolded substrates. Therefore, we verified whether HSPB8_fs mutants also sequestered chaperone-substrates, preventing their clearance. To this aim, we performed a staining with Proteostat, a dye that binds to misfolded and aggregated proteins, and we observed that the core of the HSPB8_fs aggregates was composed of misfolded proteins (Figure 3A). Next, we confirmed that the misfolded substrates were ubiquitinated, by a staining using the FK2-antibody (Figure 3B). This was further assessed by western blot analysis, in which the overexpression of HSPB8_fs mutants correlated with an increase in ubiquitinated proteins in the NP-40 insoluble fraction in presence of V5-tagged HSPB8_fs mutants (Figure 3C).

To investigate an impairment in the CASA pathway caused by the HSPB8_fs mutants, we analyzed the CASA complex activity against some of its known substrates. Since proteins with polyglutamine (polyQ) expansions are degraded by the CASA complex [17,43,46], we transiently transfected NSC34 cells with untagged HSPB8 constructs together with a FLAG-tagged polyQ stretch (polyQ74) and tested polyQ aggregation in FRA. As expected, HSPB8_WT expression caused a decrease in the levels of SDS-insoluble high molecular weight polyQ species, while the HSPB8_fs mutants exerted no significant effects on polyQ aggregation in FRA (Figure 3D). We therefore tested whether the HSPB8_fs mutants were still able to interact with CASA substrates. NSC34 transiently expressing a mCherry-tagged HTT (huntingtin) with the pathogenic polyQ tract (HTT-73Q) displayed aggregates in the nuclei, as shown, but also in the cytoplasm (Figure 3E). HSPB8_WT overexpression resulted in a decreased signal related to mCherry-HTT-73Q, suggesting CASA-mediated degradation and in agreement with previous reports [43]. Instead, HSPB8_fs mutants expression resulted in their co-aggregation with the mCherry-HTT-73Q in the cytoplasm. Comparable results were observed in cells overexpressing the GFP-tagged C-terminal fragment TDP-25, a pathogenic fragment of TARDBP/TDP-43 (TAR DNA binding protein) which mislocalizes forming cytoplasmic aggregates. Again, while expression of HSPB8_WT was associated with the clearance of GFP-TDP-25, HSPB8_fs mutants co-aggregated with the pathogenic protein (Figure 3E).

Although it is not possible to conclude whether the presence of mCherry-HTT-73Q or GFP-TDP-25 aggregates in cells expressing HSPB8_fs mutants is a consequence of HSPB8_fs dominant negative action on CASA or the HSPB8_fs mutants aggregation properties, these results suggest that HSPB8_fs mutants interfere with CASA complex activity, and that their expression correlates with the accumulation of misfolded and ubiquitinated proteins.

### HSPB8_fs mutants recruit selective autophagy receptors but do not impair the autophagic flux

**Figure 4.**
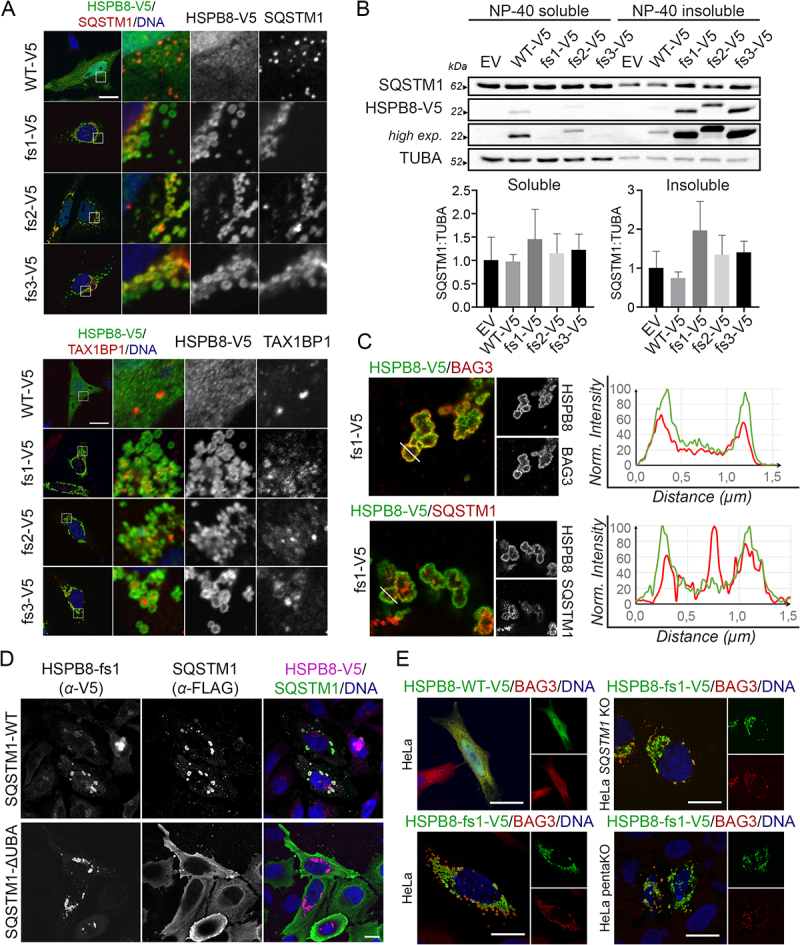
HSPB8_fs mutants recruit selective autophagy receptors but do not impair the autophagic flux. (A) Immunofluorescence analyses of HeLa cells transiently transfected with V5-tagged HSPB8 constructs (green) and stained for SQSTM1 (Top panel, red) or TAX1BP1 (Bottom panel, red). Nuclei were stained with DAPI, scale bar: 20 μm. (B) Western blot analysis of NP-40 soluble/insoluble protein extracts of HeLa transiently transfected with an empty vector or V5-tagged HSPB8 constructs. Bar graphs report mean values (± SD) of densitometry of SQSTM1 on soluble TUBA. All graphs are normalized to control cells (EV). One-way ANOVA with Tukey’s test was performed; n = 3. (C) Expansion microscopy of HeLa cells transiently transfected with V5-tagged HSPB8_fs1 (green) and stained for BAG3 (top) or SQSTM1 (bottom) (red). Normalized fluorescence intensity of the V5-tagged HSPB8 and BAG3 or SQSTM1 signals are plotted on the white line segment. The distance of the line (in µm) is corrected for the four-time-expansion of the sample. (D) Immunofluorescence analysis of HeLa cells transiently transfected with a representative V5-tagged HSPB8_fs mutant (fs1) and FLAG-tagged SQSTM1-WT or a variant lacking the UBA domain (ΔUBA). V5-tagged HSPB8 is in purple, FLAG-tagged SQSTM1 in green and nuclei were stained with DAPI, scale bar: 15 μm. (E) Immunofluorescence analysis of HeLa transiently transfected with V5-tagged HSPB8_WT or a representative HSPB8_fs mutant (fs1) (left); SQSTM1 KO and 5KO cells transiently transfected with the HSPB8_fs mutant construct (fs1) (right). V5-tagged HSPB8s are in green, BAG3 in red and nuclei were stained with DAPI, scale bar: 20 μm. The constructs were abbreviated as follows: empty vector (EV), V5-tagged HSPB8_WT (WT-V5) and HSPB8_fs mutants (fs1-V5, fs2-V5, fs3-V5).

Since we observed a general proteostasis collapse, we tested the effect of HSPB8_fs mutants on the autophagy pathway. To this end, we first evaluated the intracellular distribution of the SQSTM1 and TAX1BP1 autophagic receptors. As shown in Figure 4A, SQSTM1 and TAX1BP1 colocalized with the HSPB8_fs mutants, while in the case of the HSPB8_WT, these two autophagic receptors displayed their typical punctate pattern in the cytoplasm. Accordingly, we observed increased recruitment of the selective autophagy receptor SQSTM1 into the insoluble fraction, using western blot analysis (Figure 4B). Overall, HSPB8_fs mutants did not affect the autophagic flux, when compared to HSPB8_WT, since no alterations in SQSTM1 and LC3-II levels were observed under basal conditions, nor upon starvation and autophagy blockage using Bafilomycin A1 (**Figure S3A**).

As we previously observed that misfolded substrates resided in the inner core of HSPB8_fs mutant structures, we performed super-resolution microscopy on HeLa cells to gain insight into the ultrastructural organization of HSPB8_fs clusters. We focused on the HSPB8_fs1 as a representative mutant forming hollow aggregates. Using expansion microscopy, we confirmed the localization of V5-tagged mutant HSPB8 on the rim of such cytoplasmic structures, together with BAG3 (Figure 4C). Conversely, when we performed expansion microscopy to assess the localization of SQSTM1 relative to HSPB8, we observed that the autophagy receptor also resided in the inner core, suggesting that it may directly interact with the ubiquitinated cargo (Figure 4C). Indeed, the deletion of the ubiquitin-binding UBA domain prevented the recruitment of SQSTM1 to HSPB8 aggregates (Figure 4D).

As SQSTM1 is known to induce condensation of its client proteins [47] and HSPB8 was shown to interact with SQSTM1 upstream to BAG3 and HSPA interaction [14], we wondered whether the characteristic localization of HSPB8_fs mutants at the outside borders of the protein aggregates is the result of a SQSTM1-driven condensation reaction. Therefore, we tested whether depleting autophagy receptors would prevent the HSPB8_fs mutant from forming these protein-aggregate positive structures. To this aim, we took advantage of HeLa cells lacking SQSTM1 (HeLa *SQSTM1* KO) and HeLa cells lacking five autophagic receptors (SQSTM1, CALCOCO2/NDP52 [calcium binding and coiled-coil domain 2], TAX1BP1, NBR1 [NBR1 autophagy cargo receptor], and OPTN [optineurin]), here named as pentaKO or 5KO cells [48–50]. The *SQSTM1* KO or pentaKO cells were successfully tested for autophagic receptors protein depletion and transfected with one representative HSPB8_fs mutant (fs1) to test for HSPB8 aggregation (**Figure S3B**). Notably, despite the absence of the autophagic receptors, the selected HSPB8_fs mutant still displayed aggregation propensity indicating that these autophagy receptors are not the driving force of clustering the ubiquitinated client proteins.

### The BAG domain of BAG3 is required to facilitate clustering of HSPB8_fs mutant aggregates

**Figure 5.**
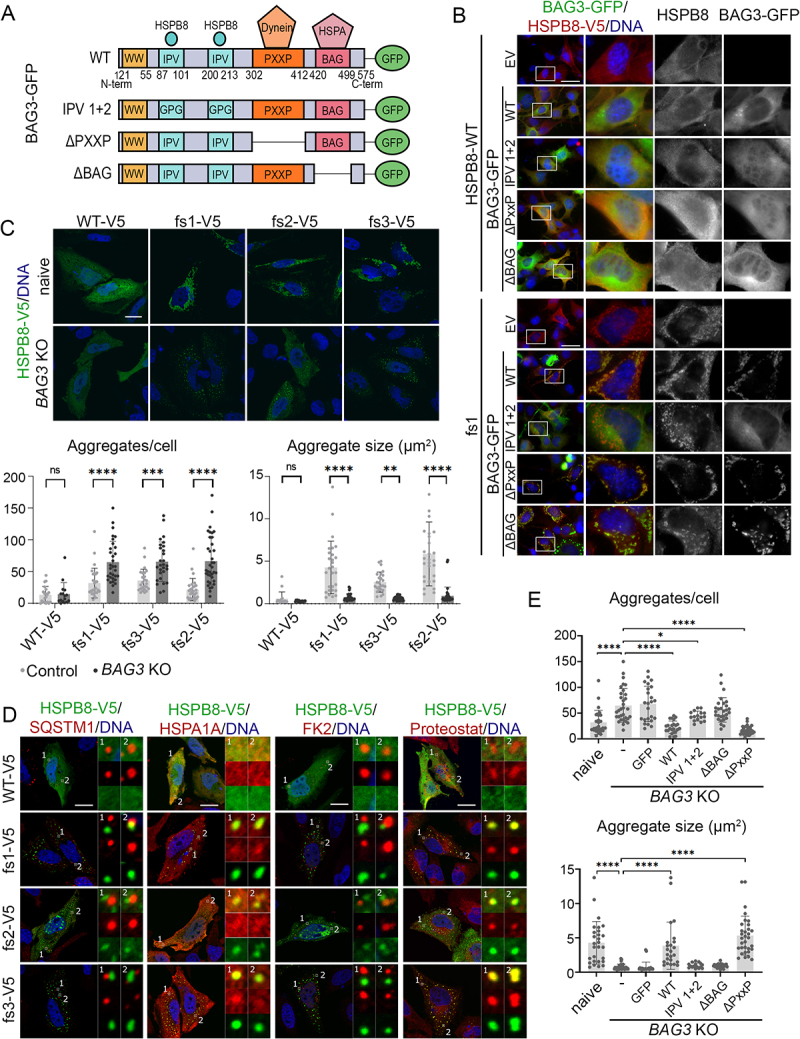
The BAG domain of BAG3 is required to facilitate clustering of HSPB8_fs mutant aggregates. (A) Schematic representation of the BAG3-GFP protein domains and functional mutants. BAG3-GFP WT has a WW domain, two IPV domains for HSPBs interaction (e.g., HSPB8), a PxxP domain for dynein motor complex interaction, a BAG domain for HSPA interaction and a GFP moiety at the C-terminus. The BAG3-GFP functional mutants used in this study are: IPV1 + 2 (GPG substitution of both IPV domains), ΔPxxP in which the PxxP is deleted, ΔBAG in which the BAG domain is deleted abolishing the HSPA interaction. (B) Immunofluorescence analysis of NSC34 cells transiently co-transfected with an empty vector (EV) or BAG-GFP constructs (WT or mutated) and with untagged HSPB8_WT or a representative HSPB8_fs mutant (fs1) constructs. Immunofluorescence was performed against HSPB8 (red), BAG3-GFP is in green, and nuclei were stained with DAPI, scale bar: 10 μm. (C) Immunofluorescence analysis of HeLa parental (naive) or HeLa *BAG3* KO cells transfected with V5-tagged HSPB8 constructs. V5-tagged HSPB8 is in green (α-V5), and nuclei were stained with DAPI, scale bar: 20 μm. On the bottom, bar graphs report the total number of aggregates per cell and the aggregate size (µm^2^) in HSPB8_WT and HSPB8_fs mutants in HeLa parental or HeLa *BAG3* KO cell lines. Each data point represents the measurement of a single cell. Two-way ANOVA with Šídák’s multiple comparisons test was performed: ** p < 0.01, *** p < 0.001, **** p < 0.0001. (D) Immunofluorescence analyses of HeLa *BAG3* KO cells transfected with V5-tagged HSPB8_WT or HSPB8_fs mutants. V5-tagged HSPB8 is in green, SQSTM1, HSPA1A, ubiquitinated substrates (FK2), or Proteostat in red and nuclei were stained with DAPI, scale bar: 20 μm. The insets show details on selected areas of the cell, displaying presence or absence of colocalization between HSPB8-V5 and the proteins of interest. (E) Graphs report the total number of aggregates per cell and the aggregate size (µm^2^) of aggregates formed by a representative V5-tagged HSPB8_fs mutant (fs1) in HeLa parental (naive) or HeLa *BAG3* KO cells transiently transfected with eGFPN1 or BAG-GFP constructs (WT or mutant). One-way ANOVA with Dunnett’s multiple comparisons test was performed: * p < 0.05, **** p < 0.0001. The constructs were abbreviated as follows: empty vector (EV), V5-tagged HSPB8_WT (WT-V5) and HSPB8_fs mutants (fs1-V5, fs2-V5, fs3-V5), untagged HSPB8_WT (HSPB8-WT) and HSPB8_fs1 mutant (fs1), GFP-tagged BAG3s (BAG3-GFP).

We next wondered whether HSPB8_fs mutant aggregation was driven by the expanded C-terminal sequence (i.e., intrinsic) or by interaction with other factors of the CASA complex (i.e., extrinsic). In fact, it has been shown that mutations in BAG3 also result in protein aggregation, which could be prevented by interfering with the interaction between BAG3 and HSPA [27]. We therefore verified whether HSPB8_fs mutant aggregation could be abolished by direct inhibition of the HSPB8 interaction with CASA members. To this aim, we focused on BAG3 as it directly binds to HSPB8, connecting it to the rest of the CASA machinery. The interaction of BAG3 with HSPB8 is mediated by two IPV motifs present in the BAG3 protein that bind a hydrophobic groove on the ACD, formed by β4 and β8 sheets of HSPB8 [44], and we evaluated whether the HSPB8_fs mutants still interacted with BAG3 using this canonical binding groove. To this end, HSPB8_WT or HSPB8_fs mutants were co-expressed with either GFP-tagged BAG3 (BAG3-GFP) WT or BAG3-GFP proteins lacking: i) the two IPV motifs (IPV 1 + 2), or ii) the PxxP (ΔPxxP) domain to block its binding to the dynein motor complex, or iii) the BAG (ΔBAG) domain to prevent its interaction with HSPA (Figure 5A). Immunofluorescence analysis performed on NSC34 cells transiently overexpressing the different BAG3-GFP constructs with the untagged HSPB8_WT or one representative mutant, the HSPB8_fs1, are shown in Figure 5B. Notably, in cells expressing HSPB8_WT, all BAG3-GFPs mainly displayed a diffuse and homogeneous intracellular distribution, with a small percentage of cells exhibiting a punctate GFP-BAG3 localization (**Figure S4A**). As expected, also the intracellular distribution of HSPB8_WT remained unaffected by the presence of the various BAG3-GFPs. On the other hand, cells expressing the HSPB8_fs1 mutant were all characterized by co-aggregation of HSPB8 and BAG3-GFP, except for the BAG3-GFP construct lacking the two IPV motifs. Indeed, while the HSPB8_fs mutant (fs1) still formed cytoplasmic aggregates, the BAG3-GFP IPV 1 + 2 remained normally diffused in the cytoplasm. In support of this result, we performed a fractionation assay based on the NP-40 soluble/insoluble protein extraction to assess the solubility of BAG3-GFP constructs in presence of HSPB8_WT or its mutant (**Figure S4B**). We found that HSPB8_WT almost completely partitioned in the soluble fraction, with only a small portion of HSPB8_WT detectable in the insoluble fraction. Instead, the HSPB8_fs mutant (fs1) was always present in the insoluble fraction. The partitioning of BAG3-GFP_WT or its deletion mutants paralleled the behavior of HSPB8_WT or HSPB8_fs mutant in all cases, except for the BAG3-GFP IPV 1 + 2. These data therefore confirm that the recruitment of BAG3 to HSPB8_fs mutants is mediated through the conventional IPV-motifs and is not resulting from nonspecific interactions with other domains of the BAG3 protein.

To test whether HSPB8_fs aggregation depends on its interaction with BAG3, we generated HeLa *BAG3* KO cells and transfected them with V5-tagged HSPB8 constructs. Western blot analysis on NP-40 soluble/insoluble protein extracts showed that BAG3 depletion was not able to restore the solubility of the HSPB8_fs mutants, since they still partitioned in the NP-40 insoluble fraction (**Figure S4C**). Using microscopy, we also examined the HSPB8_fs mutant aggregates in *BAG3* KO cells (Figure 5C). In cells lacking BAG3, clusters of HSPB8_fs mutant aggregates were more dispersed across the cytoplasm and were typically smaller in size compared to BAG3-containing cells. This suggests that the interaction of HSPB8_fs with BAG3 is necessary to gather HSPB8 aggregates in larger perinuclear deposits and is in line with the function of BAG3 to transport substrates to the MTOC [11]. As expected, HSPB8_fs structures in *BAG3* KO cells were devoid of SQSTM1 and negative for ubiquitinated protein staining, while HSPA1A mainly displayed a diffuse signal and minimally colocalized with HSPB8_fs aggregates. Instead, HSPB8_fs structures were still positive for Proteostat staining, possibly indicating the presence of entrapped misfolded substrates (Figure 5D). Interestingly, we also observed that HSPB8 fs1 and fs3 aggregates did not display the hollow core in *BAG3* KO cells, further suggesting that BAG3 aids in the enlargement and evolvement of HSPB8_fs mutant structures.

BAG3 directs misfolded proteins to aggresomes by loading them onto the dynein motor complex, which is mediated by its PxxP domain. A BAG3 mutant lacking the PxxP domain was previously shown to fail to load cargo on a dynein motor [11]. As *BAG3* KO induced a shift toward a more diffuse distribution of small HSPB8_fs aggregates, we speculated that the loss of BAG3-mediated dynein binding was the underlying cause. However, when we reintroduced GFP-tagged BAG3 in presence of HSPB8_fs1, we observed a preferential partitioning of BAG3_WT and BAG3_ΔPxxP in the insoluble fraction, accompanied by an increase in insoluble ubiquitinated proteins. Conversely, BAG3 IPV 1 + 2 mutant mainly partitioned in the soluble fraction and associated with a reduction in insoluble ubiquitinated proteins (**Figure S4D**). Surprisingly, the BAG3_ΔBAG mutant partitioned both in the soluble and insoluble fractions but did not induce an accumulation of insoluble ubiquitinated proteins. In support of this, only BAG3_WT and BAG3_ΔPxxP were able to induce the clustering into larger aggregates and could rescue the *BAG3* KO phenotype to the control cell level (Figure 5E). Together, these data show that the BAG domain plays an essential role in the CASA complex. In absence of the BAG domain, both HSPA as well as E3-ubiquitin ligases required for the ubiquitination of misfolded substrates fail to get recruited to the misfolded protein aggregates and this prevents their transport toward the perinuclear region.

Altogether, our data show that the HSPB8_fs aggregation occurs independently of other CASA complex members but that BAG3 is required for the recruitment of HSPA and E3-ubiquitin ligases to ubiquitinate the misfolded cargo and to induce their clustering at the MTOC. These data therefore suggest a strict hierarchy in the functioning of the CASA complex, with maturation and clustering of ubiquitinated cargo material at the MTOC depending on prior ubiquitination of the misfolded substrates, a step that is facilitated by the BAG domain of BAG3.

### The mutated C-terminal region and its extension are determinants for protein aggregation

**Figure 6.**
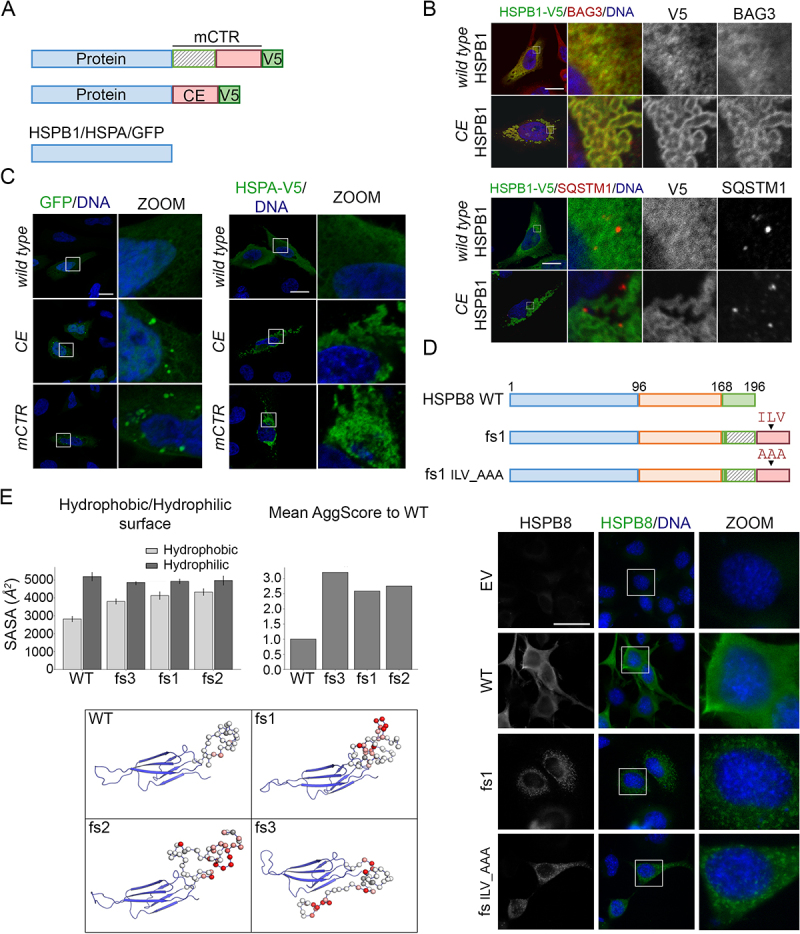
The mutated C-terminal region and extensions are determinant for protein aggregation. (A) Schematic representation of constructs used to test the aggregation propensity of the C-terminus fs sequences: the mCTR and the CE, as indicated in Figure 1A. In blue: tested proteins (HSPB1, eGFP or HSPA); in green: V5 tag. (B) Immunofluorescence of HeLa cells transiently transfected with V5-tagged HSPB1 constructs (HSPB1-WT or HSPB1-CE). Immunofluorescence analyses was performed against HSPB1-V5 (green) and BAG3 (red, top panel) or SQSTM1 (in red, bottom panel). Nuclei were stained with DAPI, scale bar: 20 μm. (C) Fluorescence microscopy on HeLa transiently transfected with GFP constructs ([GFP-]WT, [GFP-]CE or [GFP-]mCTR, on the left) and immunofluorescence of HeLa transiently transfected with V5-tagged HSPA constructs ([HSPA-]WT, [HSPA-]CE or [HSPA-]mCTR on the right). Immunofluorescence was performed against HSPA-V5 (α-V5, green). Nuclei were stained with DAPI, scale bar: 20 μm. (D) Schematic representation of HSPB8_WT and the HSPB8_fs mutant constructs (fs1): the arrowhead indicates the localization of the new ILV motif in the HSPB8_fs sequence, which was substituted by an Alanine tripeptide (AAA). Immunofluorescence analysis of NSC34 cells transiently transfected with HSPB8 constructs (HSPB8_WT, HSPB8_fs or its mutant HSPB8_fs ILV_AAA) or an empty vector. HSPB8 is in green, nuclei were stained with DAPI, scale bar: 10 μm. (E) The graphs report the mean hydrophobic/hydrophilic solvent-exposed surfaces (SASA) of HSPB8 variants calculated during MD trajectories and mean aggregation propensity score (AggScore) for HSPB8_fs mutants normalized to HSPB8_WT, computed on MD simulations medoids. At the bottom, structures of HSPB8_WT and HSPB8_fs mutants (MD simulations medoids): elongated C-termini form highly mobile disordered structures with several hydrophobic patches. Alpha carbons of the C-terminal loops are represented as spheres colored according to their aggregation propensity score (AggScore), ranging from red (high aggregation propensity) to white (low aggregation propensity).

Since HSPB8_fs mutants cause HSPB8 aggregation independently of the interaction with CASA protein members or autophagic receptors, we sought to gain insight into the mechanism of chaperone dysregulation. To this aim, we focused on the role of the mutated C-terminus in HSPB8_fs aggregation proneness, by generating constructs expressing different proteins fused with: i) the mutated CTR (mCTR, present in fs1 and fs3) or ii) the CE (present in all mutants) of the HSPB8_fs mutants (Figure 6A). First, we analyzed the behavior of HSPB1/Hsp27 (heat shock protein family B (small) member 1), another chaperone of the HSPB family, fused with the HSPB8_fs CE. HeLa cells overexpressing an HSPB1_WT construct showed a diffuse cytoplasmic distribution of the HSPB1 protein and no alteration in BAG3 or SQSTM1 localization was observed. Instead, when the HSPB1-CE construct was transfected, strong perinuclear clustering of the HSPB1 fusion protein occurred, phenocopying what we observed for HSPB8_fs mutants. Interestingly, BAG3 was recruited and colocalized with HSPB1_fs mutants, while SQSTM1 displayed no colocalization (Figure 6B). This led us to speculate that the HSPB1-CE fusion protein aggregation might be related to structural and/or functional features shared between HSPBs. We therefore decided to test this hypothesis by fusing the CTR and CE to other protein substrates, that are not part of the HSPB family. HSPA and GFP were chosen based on their different molecular weight and different relationship with CASA. Overexpression of GFP-CE or -mCTR fusion constructs was highly toxic to cells, so only a limited number of cells bearing aggregates was observed 24 h (instead of 48 h) after transfection (Figure 6C). Indeed, live imaging on transiently transfected cells revealed that, while the GFP-WT protein increased in levels over time distributing diffusely in cells, the GFP-mCTR/-CE fusion proteins rapidly formed aggregates causing cell death within a few hours after the start of the expression (**Figure S5A**). Instead, cells overexpressing the HSPA fusion constructs were viable, but the fusion construct still induced protein aggregation (Figure 6C). This suggests that this amino acid sequence is aggregation-prone and might cause proteotoxic stress, especially in those tissues with the highest HSPB8 expression.

To gain further insight into the biochemical features of the mutated HSPB8 C-terminus, we focused on its amino acid sequence properties. As recently reported [39], the CE tract generates a de novo ILV sequence, which is the canonical motif in the HSPB1 C-terminus that was shown to bind the β4/β8 groove and found to regulate both oligomer size and chaperone-client interactions [51]. To assess whether this de novo motif is responsible for the dysregulation of HSPB8, we mutated this tripeptide in HSPB8_fs1 mutant (HSPB8_fs ILV_AAA) (Figure 6D). We observed that, even in the absence of the ILV sequence, the HSPB8_fs ILV_AAA still formed cytoplasmic aggregates (Figure 6D). We then verified whether there would be other motifs hidden in the new CE, responsible for the phenotypes we observed, by performing Ala-scanning on the CE tract shared by all fs mutants (**Figure S5B**). We found that all the HSPB8-Ala proteins (Ala1-4) aggregated in a similar fashion to the parental HSPB8_fs mutant (fs2-V5), arguing against the presence of a specific motif responsible for the aggregation of HSPB8_fs mutants (**Figure S5C and S5D**).

Therefore, we performed in silico analyses via molecular dynamics (MD) simulations on the HSPB8_fs mutants, which revealed an increase in the solvent-exposed hydrophobic surface of HSPB8_fs mutants compared to HSPB8_WT, whereas the hydrophilic surface did not seem to have changed significantly (Figure 6E). Surface patch projections of hydrophobic and charged patches also confirmed the propensity of HSPB8_fs mutants to form aggregates, as pointed out by the increase of their aggregation propensity scores, which is approximately 3-fold higher than the same score for HSPB8_WT (Figure 6E). The increased aggregation propensity scores are likely due to the presence of numerous hydrophobic amino acids in the elongated C-terminus, which is predicted to remain disordered and unstructured (Figure 6E). Collectively, these results show that besides dysregulation of the CASA complex function, the C-terminus of HSPB8_fs mutants is also aggregation-prone, unrelated to the presence of a specific motif, due to the altered biochemical features induced by the de novo amino acid sequence.

### HSPB8_fs mutants impair myoblast differentiation and induce actinin alpha accumulation

**Figure 7.**
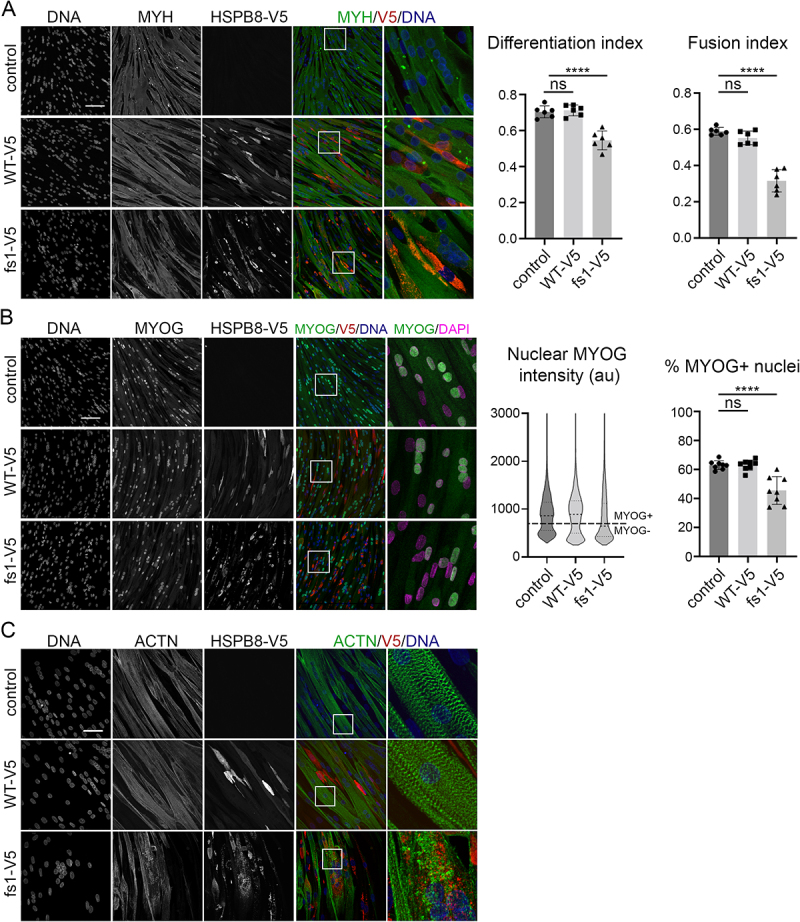
HSPB8_fs mutants impair myoblast differentiation and associate with ACTN accumulation. Panels report the immunofluorescence analyses of differentiated human control myoblasts or myoblasts stably expressing a V5-tagged HSPB8 construct. (A) V5-tagged HSPB8 (α-V5) is stained in red, MYH in green. Nuclei were stained with Hoechst33342. The differentiation index was defined as the proportion of nuclei inside MYH-positive myotubes. Multiple nuclei in MYH-positive myotubes determine the fusion index, as the proportion over all nuclei (including MYH-positive myotubes with only a single nucleus and nuclei in MYH-negative cells). Scale bar: 100 μm. (B) V5-tagged HSPB8 (α-V5) is stained in red, MYOG in green. Nuclei were stained with Hoechst33342. The violin plot represents the distribution of the MYOG mean intensity in every nucleus. A fixed intensity threshold value was applied based on two evident populations to categorize every nucleus as either MYOG-positive or MYOG-negative (value 700, indicated by the dashed horizontal line). The bar graph represents the percentage MYOG-positive nuclei. Scale bar: 100 μm. (C) V5-tagged HSPB8 (α-V5) is stained in red, the sarcomere marker ACTN is in green. Nuclei were stained with Hoechst33342. Scale bar: 50 μm. One-way ANOVA with Dunnett’s test was performed: **** p < 0.0001 (n = 1, individual data points represent data from one field of view). Data are presented as mean ± SD.

Since HSPB8 frameshift mutations have only been reported in (neuro)myopathy patients, it is likely that they primarily affect muscle tissue (Table 1). To evaluate the pathogenicity of HSPB8_fs on muscle cell biology, we differentiated human myoblasts that express HSPB8_WT or HSPB8_fs1. To this end, we transduced fully confluent myoblast cultures with lentiviral HSPB8 expression vectors and incubated the cells in differentiation medium for seven days, leading to increased cell polarization and the formation of MYH (myosin heavy chain)-positive, mono- or multinucleate myotubes (Figure 7A). To quantitatively measure the differentiation capacity, we calculated the differentiation index, defined as the ratio between the number of nuclei inside MYH-positive myotubes and the total number of nuclei. In addition, multiple nuclei inside MYH-positive myotubes determine the fusion index, as the proportion over all nuclei (including MYH-positive myotubes with only a single nucleus and nuclei in MYH-negative cells). Cells expressing HSPB8_WT exhibited comparable differentiation and fusion indexes as non-transduced control cells. In contrast, the expression of HSPB8_fs1 led to a significant decrease in both differentiation and fusion index (Figure 7A). Next, we evaluated by immunocytochemistry the presence of the transcriptional activator MYOG (myogenin), as an additional marker for myogenic differentiation. By measuring MYOG intensities inside many nuclei, MYOG-positive and MYOG-negative nuclei can be clearly distinguished into separate populations (Figure 7B). In line with previous results, HSPB8_fs1 mutant myocyte cultures contained a lower percentage of MYOG-positive nuclei compared to HSPB8_WT or non-transduced controls. Taken together, these data suggest that the HSPB8_fs mutants, upon overexpression, impair the differentiation and fusion of human myoblasts into myotubes. The specific vulnerability of muscle cells to mutant HSPB8 can be explained by the high expression levels of HSPB8 in muscle tissue, but also by the function of the CASA complex at the Z-disks, the structural elements that gather and crosslink the actin filaments from adjacent contractile units. Here, the CASA complex favors the turnover of FLNC/filamin C [5,52] and potentially other sarcomeric proteins [53]. We therefore reasoned that the accumulation and failure to degrade misfolded client proteins, as described in this manuscript, might severely impact the sarcomeric structure and function. To this end, we assessed sarcomere formation in our differentiated human myoblast cultures. Although not fully mature, control and HSPB8_WT cells displayed the typical striation pattern of sarcomeres when stained for ACTN (actinin alpha), a Z-disk marker (Figure 7C). In contrast, HSPB8_fs1 expressing cells exhibit a massive impairment in sarcomere structure organization, with loss of striation pattern and accumulations of ACTN surrounding the HSPB8_fs1 aggregates.

In summary, these results suggest that overexpression of HSPB8_fs mutants impairs the differentiation and maturation capacity of human myoblasts. In addition, the accumulation of ACTN suggests a failure in protein homeostasis at the sarcomere, in line with previous studies showing Z-disk disintegration when the CASA complex is compromised [54–56].

## Discussion

The CASA pathway operates in the recognition and removal of misfolded and aggregating substrates, by targeting them to autophagic degradation [5,6,15–18,57,58]. The CASA complex is ubiquitously expressed but it is particularly important for muscle cells and neurons, as mutations in different members of the CASA complex, including HSPB8, have been associated with neuronal and muscle diseases. However, the accumulation of a significant number of patients with fs mutations in the C-terminus of HSPB8 raises new questions about the underlying pathomechanisms, as these different fs mutations causing the production of HSPB8 proteins sharing the same C-terminal elongation have not been reported in other HSPBs [34,39,59–61]. Here, we demonstrate that the expression of HSPB8_fs mutants is associated with their misfolding and cytoplasmic accumulation into aggregated species. Notably, while HSPB8_fs mutant aggregation is apparently in contrast with a previously suggested HSPB8 haploinsufficiency mechanism [40], the loss of HSPB8 protein and its functions may not be sufficient alone to induce muscle diseases. Indeed, it has been reported that *Hspb8* KO in a mouse model does not associate with severe myopathic features [54]. Although the HSPB8 mutant aggregation hypothesis seems in conflict with the evidence that shows no elongated HSPB8_fs protein in patients derived samples, it might be also possible that cells are able to cope with HSPB8_fs mutants. For instance, cells might boost quality control systems that operate in HSPB8 downregulation through mRNA or protein degradation. However, in cells that rely on high HSPB8 expression for physiological maintenance or in response to stress, such as muscle cells, HSPB8_fs mutants might be able to escape quality control systems and accumulate into cytoplasmic aggregates. This model explains the absence of elongated HSPB8_fs mutants in patients’ fibroblasts, in which HSPB8 expression is physiologically low, but supports the observation of HSPB8-positive puncta in patients’ fibroblasts upon stress [38]. Thus, while detrimental effects caused by HSPB8_fs mutants might not be observed on several cell type characterized by low HSPB8 expression, pathogenic consequences of HSPB8_fs can be detected in muscle tissues. In support to this notion, our findings on muscle cell models suggest that HSPB8_fs associated with impaired differentiation and sarcomeric disorganization, with signs of sarcomeric protein ACTN accumulation.

Other evidence suggests that HSPB8 aggregation might be a pathological mechanism at the basis of neuromyopathy. Indeed, previous studies on K141 substitutions revealed HSPB8 mutant aggregation in cell and animal models [34,54,62,63] and similar histological defects in muscle biopsies from patients, consisting in myofibrillar pathology with rimmed vacuoles and dystrophic features [33,37]. Also, our findings are in line with previous observations on the HSPB8 partner BAG3. Indeed, others and we have shown that BAG3 variants carrying neuromyopathy-causative mutations display an aggregation-prone behavior and relocate the other CASA complex components into aberrant aggresomes [27,45]. Similarly, we also observed the relocation of CASA members in HSPB8_fs mutant cytoplasmic sub-compartments together with autophagic receptors. However, in the case of BAG3, its aggregation can be rescued by abrogating the interaction with its chaperone HSPA [27]. Conversely, HSPB8_fs mutant dysregulation occurs upstream and independently of the CASA complex but results in similar defects in CASA-mediated disposal of substrates (e.g., polyQ substrate). Noteworthy, the failure of the protein quality control system caused by HSPB8_fs mutants is consistent with the accumulation of autophagic markers (e.g., SQSTM1) reported in patients’ fibroblasts and biopsies [33,38].

Our analyses support the hypothesis that the mCTRs of HSPB8 are intrinsically disordered and prone to aggregation. Interestingly, the CTR is highly variable among HSPBs, except for the IxI/V motif present in some members of the same protein family [1]. HSPB8 naturally lacks this motif and replacing its C-terminus with that of HSPB1 (harboring an IPV motif) results in a partially insoluble protein and the loss of its chaperone activity toward polyQ-expanded huntingtin [44]. In contrast, the substitution of this IPV motif restores solubility and chaperone activity, indicating that introducing an IxI/V motif in HSPB8 interferes with its structure and function. In addition, it was recently shown that the P182L mutation in the IxI/V motif of HSPB1 results in larger HSPB1 oligomers and aberrant binding to interacting proteins, underscoring the important regulatory role of the IxI/V motif in modulating chaperone-client interactions [51]. However, we were unable to identify motifs driving aggregation of HSPB8_fs, suggesting that other factors, such as hydrophobic patches rather than a specific sequence of amino acids, might explain its aggregation propensity. Indeed, despite the amino acidic diversity, HSPB CTRs display high flexibility due to the presence of highly polar amino acids and this is thought to exert a solubilizing activity [64,65]. The substitution of the C-terminus with a novel, more hydrophobic C-terminal peptide sequence may therefore have a dual impact on HSPB8_fs. On one hand, it could reduce the solubility of the chaperone due to the loss of the solubilizing effect of the C-terminus. On the other hand, and perhaps more in line with the effect of other disease-causing HSPB mutations, it could also dysregulate the chaperone activity of HSPB8. As revealed by structural studies, the N- and C-termini of several HSPBs can fold back and bind grooves on the surface of the ACD. This groove binding occurs dynamically with only a fraction of the termini bound into grooves at any given time [66]. This is thought to provide a selection mechanism for incoming client proteins, that would have to compete with the N or C-termini for binding the catalytic ACD domain [51]. As such, the N and C-termini play an important role in the chaperone activity, possibly providing stringency during client-binding but also ensuring timely client release by potentially competing the bound client proteins off again. The new peptide sequence could therefore prevent the C-terminus from fitting into the grooves on the ACD, thereby leaving the client binding grooves continuously exposed. The accumulation of hydrophobic, ubiquitinated substrates in the core of the HSPB8 structures in cells expressing HSPB8_fs mutants would be consistent with this hypothesis, as it suggests that the chaperone activity of HSPB8 might indeed be impaired. The formation of long-lasting chaperone-client complexes due to the failure of releasing client proteins would explain why HSPB8_fs mutants are at the rim of dense misfolded protein clusters, ultimately preventing their efficient degradation by the autophagy machinery.

Noteworthy, several mutations causing a truncation, modification, or elongation of the C-terminal domains have been described in other HSPBs (recently reviewed in [67]). Although many of these mutations still lack a deep characterization, for some mutants there have been comparable biochemical alterations described or predicted [68–70]. This might be indicative that insolubility and chaperone dysregulation are common features of C-terminally mutated HSPBs and associate with a shared gain of proteotoxic function. However, distinct intracellular pathogenic consequences, based on HSPBs specific functions and tissue expression, should be also investigated. For instance, HSPB8 mutant toxicity has implications for the CASA pathway and, likely, for other specific activities, such as the granulostasis maintenance or cytosolic integrated stress response, which are worth investigating [13,71–73].

A peculiar feature of HSPB8 with respect to the other HSPBs is that only HSPB8 seems to have accumulated a large number of fs mutants that do not result in premature stop codons but instead encode for longer polypeptide sequences, which are stably expressed in cells. Sequence analysis reveals that fs mutants lead to the formation of this toxic protein sequence from as early as amino acid 156; however, given that this would still reside in the last β-strand of the ACD, it seems likely that such variants are degraded in cells and disease-causing frameshifts would probably only arise from amino acid 170 onwards. This insight may help to guide clinicians when they encounter new genetic variants in HSPB8.

Inspection of the HSPB8 sequence across species reveals that this C-terminal vulnerability is a rather recent evolutionary feature. The evolution of *Euarchontoglires*, resulting in the separation of the clades of primates and glires, resulted in a 1-bp deletion in the primate 3ʹUTR, making it susceptible to the production of the toxic peptide sequence. In fact, the 3ʹUTR sequence just downstream of the stop codon has been evolutionarily active with a number of substitutions, insertions, and deletions, ultimately resulting in a sequence that has become evolutionarily stable in higher primates (conserved from macaques to humans – with the exception of gibbons), but at the cost of carrying a genetic susceptibility for frameshift mutations in the C-terminus of HSPB8 that results in the production of the toxic peptide sequence.

To conclude, the HSPB8_fs mutations related to neuromuscular diseases alter HSPB8 solubility, causing its accumulation into cytoplasmic aggregates and relocation of the CASA complex factors. This leads to weakening of the CASA and an increase in insoluble ubiquitinated proteins, ultimately causing a general failure in proteostasis maintenance.

## Study limitations

The findings of this study have some limitations. Although we fully assessed the behavior and effects of HSPB8_fs mutations on the CASA complex and proteostasis, their functional consequences on muscle and neuronal cells still need to be better dissected to establish how these mutations directly cause cell death. Unfortunately, the lack of data from patient-derived samples prevented us to validate our hypothesis on the aggregation of the HSPB8_fs mutants. Indeed, we attempted to perform immunohistochemistry on muscle biopsies from a patient, that were collected and described in 2014 [33]. Unfortunately, the remaining muscle tissue left was no longer representative because just a few fibers were present among fatty-fibrotic tissue. Despite this, immunohistochemistry results display that muscle fibers had increased staining for HSPB8, but are not shown because of the inadequate quality. We are aware that additional studies from patient-derived cell models, such as induced pluripotent stem cells to differentiate to muscle or neuronal cell models, or animal models, should be developed to better address the role of HSPB8_fs mutants in muscle pathology and assess the functional consequences on muscle physiology. Future studies oriented in this direction will bring additional knowledge to potential therapeutic strategies.

## Materials and Methods

### Plasmids

The following plasmids were used: pCDNA3 (Invitrogen, V790-20) is an empty vector (EV) used as a control; pLenti6/HSPB8-V5 encoding V5-tagged HSPB8_WT was previously described [45]; pLenti6/HSPB8-V5 p.P173Sfs*43 (fs1-V5), p.T194Sfs*23 (fs2-V5), Q170Gfs*45 (fs3-V5) were obtained by in vitro mutagenesis of HSPB8-3UTR (synthesized by GenScript USA Inc) to introduce the corresponding mutations, followed by Gateway cloning according to the companies’ protocol using BP- and LR-reaction enzymes (Thermo Fisher Scientific, 11789020 and 11791020) and pDONR221 and pLenti6/V5 vectors (Thermo Fisher Scientific, 12536017 and V49610) (Table 2). All plasmids were sequence verified with Sanger sequencing; pCi-HSPB8_WT encoding the HSPB8_WT was previously described [16]; pCi-HSPB8_fs mutants p.P173Sfs*43 (fs1), p.T194Sfs*23 (fs2), p.Q170Gfs*45 (fs3), p.P173Sfs*43 ILV_AAA (fs ILV_AAA) were obtained by subcloning the predicted mutated sequence in pCi-HSPB8_WT vector, using *Sal*I and *Apa*I restriction enzymes and T4 ligase. All the enzymes were purchased from NEB (New England Biolabs). The sequencing of the obtained plasmids was performed by Eurofins Genomics service. The pCDNA 5/TO FLAG-tagged polyQ encodes for a FLAG-tagged polyQ expansion (74Q) and was obtained by cloning the polyQ tract from pFRT-TO-FLAG-HDQ74 to pCDNA 5/TO (with modified multicloning site) using *Hind*III and *Not*I restriction enzymes and T4 ligase (New England Biolabs), pFRT-TO-FLAG-HDQ74 was obtained from the pFRT-TO-EGFP-HDQ74 plasmid [74]; pEGFP-TDP-25, which encodes the TARDBP/TDP-43 C-terminal fragment (25 kDa) fused with GFP protein at the N-terminus, was kindly provided by Dr. Leonard Petrucelli (Department of Neuroscience, Mayo Clinic, Jacksonville, FL 32224, USA) [75]; pLenti6/HSPB1-V5 encoding a V5-tagged HSPB1 WT was previously described [76] and the pLenti6/HSPB1-V5 carrying the CE was obtained by subcloning HSPB1-CE (synthesized by GenScript USA Inc) using Gateway cloning following the companies’ protocol (Thermo Fisher Scientific); EGFP- or HSPA-mCTR/CE fusions were generated by In-Fusion cloning (Takara Bio Inc). Briefly, fragments were PCR amplified containing complementary overhangs and combined with a linearized pUC19 vector and the In-Fusion enzyme master mix. Primers were designed using the In-Fusion Cloning Primer Design Tool (https://www.takarabio.com/learning-centers/cloning/primer-design-andother-tools). Next, Gateway cloning (following manufacturer’s protocol) was used to generate V5-tagged constructs (including EGFP and HSPA WT) in the pLenti6/V5 backbone; pBAG3-GFP wild-type (WT) and mutants IPV 1 + 2, ΔPxxP, ΔBAG encode for GFP-tagged BAG3 WT or functional mutants and were kindly provided by Dr. Josée N. Lavoie (Université Laval, Québec, Canada) and described previously [77]. Constructs used for Ala-scanning were obtained from pLenti6/HSPB8-V5_fs2 by using in vitro mutagenesis (IVM) (Table 2). The IVMs were performed either with the KAPA HiFi HotStart PCR kit (Roche Diagnostics Belgium, 07958897001) followed by *Dpn*I digestion (Thermo Fisher Scientific, FD1704), or by the QuikChange Lightning Site-Directed Mutagenesis Kit (Agilent Technologies, 210518).

**Table 2.** List of primers used for cloning.

HSPB8_fs MUTANTS (pLenti6/V5)		
NAME	PRIMER	purpose
HSPB8-3UTR_GW_Fw	GGGG ACAAGTTTGTACAAAAAAGCAGGCT ACCATG GCTGACGGTCAGATGCCCT	GW
HSPB8-3UTR_GW_Rv	GGGG ACCACTTTGTACAAGAAAGCTGGGTC GAGAAGCCCTAGGGTTGGGGACA	GW
HSPB8_515dupC_IVM_Fw (fs1-V5)	CATCGAAGCTCCCCAGGTCCCCTCCTTACTCAACATTTGGAG	IVM
HSPB8_515dupC_IVM_Rv (fs1-V5)	CTCCAAATGTTGAGTAAGGAGGGGACCTGGGGAGCTTCGATG	IVM
HSPB8_508-9delCA_IVM_Fw (fs3-V5)	TCTGCTGATCATCGAAGCTCCCGGTCCCTCCTTACTCAACATT	IVM
HSPB8_508-9delCA_IVM_Rv (fs3-V5)	AATGTTGAGTAAGGAGGGACCGGGAGCTTCGATGATCAGCAGA	IVM
HSPB8_577dupGTCA_IVM_Fw (fs2-V5)	CCAGGACAGCCAGGAAGTCAGTCACCTGTACCTGAGATGCCAGTACTG	IVM
HSPB8_577dupGTCA_IVM_Rv (fs2-V5)	CAGTACTGGCATCTCAGGTACAGGTGACTGACTTCCTGGCTGTCCTGG	IVM
HSPB1-3UTR-B8_GW_Fw	GGGG ACAAGTTTGTACAAAAAAGCAGGCT ACCATG ACCGAGCGCCGCGTCCCCTT	GW
Ala1 Fw	CCCAGGACAGCCAGGAAGTCGCGGCCGCGGCTGCCGCGTGCCAGTACTGGCCCATCCT	IVM
Ala1 Rv	AGGATGGGCCAGTACTGGCACGCGGCAGCCGCGGCCGCGACTTCCTGGCTGTCCTGGG	IVM
Ala2 Fw	AAGTCAGTCACCTGTACCTGGCGGCGGCCGCCGCCGCGATCCTTGTTTTGTCCCCAAC	IVM
Ala2 Rv	GTTGGGGACAAAACAAGGATCGCGGCGGCGGCCGCCGCCAGGTACAGGTGACTGACTT	IVM
Ala3 Fw	ACCTGAGATGCCAGTACTGGGCGGCCGCCGCGGCGGCACCAACCCTAGGGCTTCTCGA	IVM
Ala3 Rv	TCGAGAAGCCCTAGGGTTGGTGCCGCCGCGGCGGCCGCCCAGTACTGGCATCTCAGGT	IVM
Ala4 Fw	ACTGGCCCATCCTTGTTTTGGCCGCGGCGGCCGCCGCTGCGGACCCAGCTTTCTTGTACAA	IVM
Ala4 Rv	TTGTACAAGAAAGCTGGGTCCGCAGCGGCGGCCGCCGCGGCCAAAACAAGGATGGGCCAGT	IVM

Primers used for generating pLenti6/V5 plasmids. GW = Gateway cloning; IVM = in vitro mutagenesis.

### Cell cultures

HeLa cells (American Type Culture Collection, CCL-2) were grown in MEM medium (Life Technologies, 11095080) completed with L-glutamine 1 mM (EuroClone, ECB3004D), penicillin G 100 U/ml (SERVA, 31749.04), streptomycin 100 U/ml (Sigma-Aldrich, S9137-25 G) and 10% fetal bovine serum (FBS; Gibco, 10270106). Murine Neuroblastoma x Spinal Cord 34 (NSC34, provided by Dr Neil Cashman, University of British Columbia, Canada) were grown in high glucose DMEM (EuroClone, ECB7501L) completed with L-glutamine 1 mM (EuroClone, ECB3004D), penicillin G 100 U/ml (SERVA, 31749.04), streptomycin 100 U/ml (Sigma-Aldrich, S9137-25 G) and 5% FBS (Sigma-Aldrich, F7524). HEK293T cells stably expressing the V5-tagged HSPB8 (HEK293T-HSPB8-V5) were obtained by lentiviral infection and grown as previously described [45]. HeLa *SQSTM1* KO and pentaKO (5KO) cells were kindly provided by Dr. Christian Behrends (Ludwig-Maximilians-Universität München, München, Germany) and Dr. Michael Lazarou (Monash University, Melbourne, Victoria, Australia; Walter and Eliza Hall Institute of Medical Research, Parkville, Victoria, Australia), respectively, and described [48,49]. Cells were grown in DMEM high glucose GlutaMAX (Gibco, 61965026) supplemented with 10% FBS (Gibco, 10270106), 1% sodium pyruvate (Gibco, 11360070) and 1% penicillin-streptomycin (Gibco, 15140122). All cell lines were maintained at 37°C, 5% CO_2_.

### Generation of CRISPR-Cas9 knockout (KO) cells

HeLa *BAG3* KO cells were generated as previously described [78]. In brief, 3 guide RNAs (gRNAs) targeting the BAG3 coding sequence were designed using the CRISPick tool (https://portals.broadinstitute.org/gppx/crispick/public) and selected based on their specificity score and number of off-targets. The gRNAs were ordered as phosphorylated primers from IDT (www.idtdna.com) and cloned into the pSpCas9(BB)-2A-Puro (PX459) plasmid (Addgene, 62988, from Feng Zhang). HeLa cells were transfected in 6-well multiwell with the PX459 plasmids using PEI MAX 40 K (PolySciences Europe, 24765–1) and puromycin was added (1 µg/ml) for 72 h. Surviving cells were then serially diluted in a 96-well plate to isolate single colonies. After expansion of the single colonies, complete KO of *BAG3* was assessed by western blotting and the presence of premature stop codons was verified by Sanger sequencing.

### Human myoblast HSPB8-V5 generation and differentiation

For lentiviral production, HEK293T cells were transiently transfected with packaging (pCMV dR8.91), envelope (pMD2-VSV), and pLenti6/V5-HSPB8 (WT or mutant) plasmids using PEI MAX 40 K (PolySciences Europe, 24765–1). After 48 h, the virus-containing supernatant was collected from the HEK293T cells, filtered through a 0.45-μm filter (Millipore, SLHV033RB), and used to infect immortalized human myoblasts (Institute of Myology, AB678C53Q; 1:5 dilution). Virus medium was removed 24 h after transduction and replaced by fresh medium. Cells were grown in Skeletal Muscle Cell Growth Medium (PromoCell, C-23060). For differentiation of human myoblasts, cells were seeded at 20,000 cells/cm^2^ on 8-well chamber slides (Ibidi, 80826) coated with Geltrex (Life Technologies, A1413302). When confluent, the medium was replaced with virus-containing medium (1:500 dilution) as described above. After 24 h, virus medium was removed and replaced by differentiation medium [high glucose DMEM (Gibco, 41965039) and 2% horse serum (Gibco, 16050–122)]. Medium was changed every other day until day 7.

### Transfection

The day before transfection, cells were seeded at the following cellular densities: HeLa cells: 140,000 cell/ml in a 24-wells multiwell for immunofluorescence, in a 12-wells multiwell for western blot and filter retardation assay (FRA); NSC34: 90,000 cells/ml in a 12-wells multiwell for western blot and FRA, 70,000 cells/ml in a 24-wells multiwell for immunofluorescence. Cells were transfected using Lipofectamine3000/P3000 reagent (Invitrogen, L3000001) or PEI MAX 40 K (PolySciences Europe, 24765–1) following the manufacturers’ instruction. To test autophagy flux, the medium was replaced with serum-free medium for 24 h (starvation) and additionally treated with bafilomycin A1 10 nM (Sigma-Aldrich, B1797) for 3 h, before sample collection.

### Western blot and filter retardation assay

Forty-eight hours after transfection, cells were harvested and centrifuged 5 min at 100 x g at 4°C. Cell pellets were then lysed in NP-40 lysis buffer (150 mM NaCl [Sigma-Aldrich, S3014], 20 mM TrisBase [Sigma-Aldrich, T1503], Nonidet P-40 0.5% [NP-40; Sigma-Aldrich, 98379], 1.5 mM MgCl_2_, glycerol 3% [Sigma-Aldrich, G5516], pH 7.4) added with protease inhibitors cocktail (Sigma-Aldrich, P8340) and 1 mM DTT (Merck Millipore, 11474). Proteins were quantified with bicinchoninic acid (BCA) assay (Cyanagen, QPRO-BCA kit Standard, PRTD1). For NP-40 soluble/insoluble fractionation, samples were prepared by diluting 20–25 µg of total proteins in the same volume of NP-40 buffer. Then, samples were centrifuged at 16,100 x g for 15 min at 4°C. Supernatants were collected and pellets resuspended in the same volume of NP-40 lysis buffer and sonicated. For western blot analyses, 20 µg of HeLa or 25 µg of NSC34 protein lysates added with sample buffer (0.6% Tris, 2% SDS [Sigma-Aldrich, L3771], 10% glycerol [Sigma-Aldrich, G5516], 5% β-mercaptoethanol [Sigma-Aldrich, M3148], pH 6.8) and heated to 100°C for 5 min, were loaded on SDS-polyacrylamide gels and electrophoresis was performed. A Trans-Blot Turbo system (Bio-Rad Laboratories, 1704150) was used to electro-transfer proteins to 0.45-µm nitrocellulose membranes (Amersham™ Protran®, GEH10600003). Alternatively, protein extracts were supplemented with NuPAGE LDS sample buffer (Life Technologies, NP0007) and DTT (Thermo Fisher Scientific, 20290) and loaded on NuPAGE gels (Life Technologies) and transferred with a Hoefer TE22 Mighty Small Transfer Tank. To detect insoluble species by FRA, 3 or 6 μg of total protein lysates in NP-40 buffer were filtered through a 20% MeOH-treated 0.2-µm cellulose acetate membrane (Whatman GE Healthcare, GEH10404180), followed by washing with NP-40 buffer. For polyQ aggregates detection, 10 µg of protein lysates prepared in 1:1 NP-40 buffer:denaturation buffer (4% SDS [Sigma-Aldrich, L3771], 100 mM DTT [Merck Millipore, 11474] in water) were heated for 5 min at 95°C and filtered, followed by washing with a solution of 0.2% (w:v) SDS (Sigma-Aldrich, L3771) in water. At the end of the procedure, all FRA membranes were treated with 20% MeOH. Western blot and FRA membranes were incubated with a blocking solution of 5% nonfat dried milk (BioBasic, NB0669) in TBS-Tween (20 mM TrisBase [Sigma-Aldrich, T1503], 140 mM NaCl [Sigma-Aldrich, S3014], pH 7.6 and 0.01% Tween 20 [Sigma-Aldrich, P1379]) for 1 h and then incubated with primary antibodies diluted in the same solution overnight. Then, membranes were washed three times in TBS-Tween for 10 min, incubated with the peroxidase-conjugated secondary antibodies and detected with enhanced chemiluminescent (ECL) detection reagent (Cyanagen, ECL Westar Antares XLS142 and ETA C Ultra 2.0 XLS075). Images were acquired using a Chemidoc XRS System (Bio-Rad Laboratories). For western blot and FRA analyses, optical densities of the bands were analyzed using Image Lab Software (Bio-Rad Laboratories). All antibodies used for western blot in this study are listed in Table 3.

**Table 3.** List of antibodies used in this study.

Antibody	Host species	Dilution		Source
		WB	IF	
V5	Rabbit polyclonal	1:1,000	1:500	Cell Signaling Technology, 13202
V5	Mouse monoclonal		1:500	Life Technologies, R960-25
HSPB8	Rabbit polyclonal	1:500		Cell Signaling Technology, 3059
HSPB8	Rabbit polyclonal		1:500	Thermo Fisher Scientific, PA5-76780
TUBA4A/ α-tubulin	Mouse monoclonal	1:2,000		Sigma-Aldrich, T6199
ACTB/β-Actin	Mouse monoclonal	1:5,000		Sigma-Aldrich, A5541
BAG3	Rabbit polyclonal	1:2,000		Abcam, ab47124
BAG3	Rabbit polyclonal		1:500	Proteintech Group, 10599-1-AP
HSPA1A/HSP70-HSPA8/HSC70	Mouse monoclonal	1:500		Santa Cruz Biotechnology, sc-24
HSPA1A/HSP70	Mouse monoclonal	1:1,000	1:50	Enzo Life Sciences, ADI-SPA-810
STUB1/CHIP	Rabbit polyclonal	1:1,000		Calbiochem, PC711
STUB1/CHIP	Rabbit polyclonal		1:100	Proteintech Group, 55,430-1-AP
Ubiquitin	Mouse monoclonal	1:500		Santa Cruz Biotechnology, sc-8017
Ubiquitinated proteins (FK2)	Mouse monoclonal		1:500	Merck Millipore, 04–263
SQSTM1/p62	Rabbit polyclonal	1:2,000		Sigma-Aldrich, P0067
SQSTM1/p62	Mouse monoclonal	1:2,000	1:500	Abcam, ab56416
FLAG	Mouse monoclonal	1:1,000		Sigma-Aldrich, F7425
PolyQ (3B5H10)	Mouse monoclonal	1:1,000		Sigma-Aldrich, P1874
TAX1BP1	Rabbit monoclonal	1:1,000	1:100	Cell Signaling Technology, 5105
CALCOCO2/NDP52	Rabbit polyclonal	1:1,500		Abcam, ab151256
NBR1	Rabbit polyclonal	1:200		Abcam, ab219862
OPTN	Rabbit polyclonal	1:200		Abcam, ab23666
RB1CC1/FIP200	Rabbit monoclonal	1:1,000		Cell Signaling Technology, 12436
GFP	Mouse monoclonal	1:1,000		Immunological Sciences, MAB-94345
GFP	Rabbit polyclonal	1:1,000		Abcam, ab290
TOMM20	Rabbit monoclonal		1:100	Abcam, ab186734
KDEL	Rabbit monoclonal		1:100	Abcam, ab176333
MAP1LC3B/LC3	Rabbit polyclonal		1:200	Sigma-Aldrich, L7543
LAMP1	Mouse monoclonal		1:200	Santa Cruz Biotechnology, sc-20011
ACTN/α-actinin, sarcomeric	Mouse monoclonal		1:250	Sigma-Aldrich, A7811
MYOG	Mouse monoclonal		1:50	Santa Cruz Biotechnology, sc-12732
MYH (myosin heavy chain)	Mouse monoclonal		1:50	R&D systems, MAB4470
**II antibody**				
anti-rabbit IgG-HRP	goat	1:10,000		Jackson ImmunoResearch Laboratories, Inc., 111–035-003
anti-mouse IgG-HRP	goat	1:10,000		Jackson ImmunoResearch Laboratories, Inc., 115–035-003
anti-rabbit 594 Alexa Fluor	goat		1:1,000	Life Technologies, Thermo Fisher, A-11012
anti-rabbit 488 Alexa Fluor	goat		1:1,000	Life Technologies, Thermo Fisher, A-11008
anti-rabbit 594 Alexa Fluor	donkey		1:500	Life Technologies, Thermo Fisher, A-21207
anti-mouse 488 Alexa Fluor	donkey		1:500	Life Technologies, Thermo Fisher, A-21202

### Fluorescence microscopy and immunofluorescence

Cells were fixed with 4% paraformaldehyde for 20 min at room temperature or with a 1:1 solution of 4% paraformaldehyde and 4% sucrose in 0.2 M PB (0.06 M KH_2_PO_4_, 0.31 M Na_2_HPO_4_, pH 7.4) for 25 min at 37°C and then washed with phosphate-buffered saline (PBS; Sigma-Aldrich, P4417) solution 3 times for 5 min. Cells were permeabilized and nonspecific sites blocked using a solution of 0.1% Triton X-100 (Sigma-Aldrich, X100), 1% bovine serum albumin (BSA; Sigma-Aldrich, A9418) and 10% FBS (Gibco, 10270106) in PBS for 40 minutes at room temperature (RT). Then, the primary antibodies diluted in 0.1% BSA in PBS, were incubated overnight at 4°C. The following day, primary antibodies were removed, and cells washed with 0.1% BSA in PBS. Then, secondary antibodies, diluted in 0.1% BSA in PBS were incubated for 1 h at RT. Three washing steps with PBS, with the middle one containing DAPI (Sigma-Aldrich, D9542) or Hoechst33342 (Life Technologies, H3570) to stain nuclei were made. Staining with the PROTEOSTAT® Aggresome detection kit (Enzo Life Sciences, ENZ-51035) was performed according to the manufacturer’s protocol, by introducing an extra 30 min incubation with the dye after the secondary antibody incubation. Lipid Spot 488 was used following the manufacturers’ instructions (Biotium, 70069). Coverslips were then mounted with MOWIOL or DAKO fluorescence mounting medium (Dako-Agilent, S3023) onto slides. Confocal microscopy images were captured and processed using a Zeiss LSM700 laser scanning confocal microscope or LSM510 Meta system confocal microscope (Zeiss) (63×/1.4 NA PlanApochromat objective, 40x/1.3 NA Plan-Neofluar objective, 20x/0.8 NA Plan-Apochromat objective) using the Aim 4.2 software (Zeiss) and analyzed using ImageJ. Fluorescence microscopy was performed with an Axiovert 200 microscope (63×/1.4 NA PlanApochromat objective) (Zeiss) with a photometric CoolSnap CCD camera (Robber Scientific) using the Metamorph software (Universal Imaging). All antibodies used for immunostainings in this study are listed in Table 3.

### Fluorescence recovery after photobleaching (FRAP)

HeLa cells were transfected with HSPB8-GFP wild-type or mutant constructs and imaged 48 h after transfection in a μ-slide 8-well (Ibidi, 80826) in FluoroBrite DMEM medium (Life Technologies, A1896701) supplemented with 10% FBS and 4 mM L-glutamine at 37°C and 5% CO_2_. FRAP measurements were performed on a Zeiss LSM700 laser scanning confocal microscope using a Plan Neofluar 40x/1.3 NA objective. Image sequences (512 x 512 pixels, 142 nm/pixel) were acquired at 1 frame per second for 90s. Five pre-bleach sequences preceded photobleaching (20 x 20 pixel region at 100% of a 5 mW 488 nm laser for 1.25 s). FRAP sequences were recorded from at least 3 cells per genotype and intensities in the bleached region were measured with the Fiji distribution of ImageJ [79] and the normalized intensities were plotted over time. For free diffusing HSPB8-GFP in wild-type cells, bleach regions were taken either in the cytosol or in the nucleus. In mutant cells, the region was selected to bleach (part of) the HSPB8-GFP in aggregates.

### Expansion microscopy

For expansion microscopy, we adapted the protocol by Chozinski et al. [80]. Cells were grown on standard cover glasses (12 mm #1.5), fixed for 20 min at room temperature in 3.2% paraformaldehyde (Thermo Fisher Scientific, 28906) and 0.1% glutaraldehyde (Laborimpex, 16360). After reduction in sodium borohydride (Sigma-Aldrich, 452882) for 5 min, we proceeded with a standard immunofluorescence staining protocol (as described above). We used anti-V5 (Life Technologies, R960-25), anti-SQSTM1 and anti-BAG3 for the immunostainings. The standard immunofluorescence staining protocol was followed by crosslinking with 0.25% glutaraldehyde (Laborimpex, 16360) in PBS for 10 min. The samples were embedded in a gel solution containing 2 M NaCl (Sigma-Aldrich, S9625), 2.5% (w:w) acrylamide (Bio-Rad Laboratories, 1,610,140), 0.15% (w:w) N,N’-methylenebisacrylamide (Sigma-Aldrich, 146072), 8.625% (w:w) sodium acrylate (Sigma-Aldrich, 408220) in PBS. Polymerization of this reaction was activated by addition of TEMED (Santa Cruz Biotechnology, sc-29111) and APS (Thermo Fisher Scientific, 17874) after which the solution was quickly added on top of the glass coverslip. After the gels had polymerized at RT, the gels were incubated for 30 min at 37°C in a digestion buffer containing 8 U/ml Proteinase K (Thermo Fisher Scientific, 25530049). Cover glasses were removed from underneath the digested gels. The gels were then incubated in high volumes (>30 ml) of distilled water, exchanged at least 5 times to obtain the maximal expansion of the gels. The expanded gels (4x expansion) were trimmed and positioned in a 50-mm diameter glass bottom dish (WillCo Wells, GWST-5040) and immobilized using 2% UltraPure LMP agarose (Invitrogen, 16520). Images were acquired on a Zeiss LSM700 with Plan-Apochromat 63x/1.40 objective.

### Correlative light and electron microscopy (CLEM)

HeLa cells were seeded in 6-well plates at a density of 300,000 cells/well and the next day transfected with a mixture of GFP- and V5-tagged (both 1.5 µg) constructs as described before. 24 h after transfection, cells were replated to gridded 35 mm glass-bottom dishes (MatTek P35g-1.5–14-C-GRID) at a density of 75,000 cells/dish. The next day, prior to fixation, cells were incubated in serum-free medium containing 250 nM MitoTracker Deep Red (Invitrogen, M22426) for 30 min at 37°C. Cells were subsequently fixed with 4% paraformaldehyde for 20 min at RT, washed 3 times with PBS and imaged using a LSM700 laser scanning confocal microscope. Overview images (640 µm x 640 µm; 20x/0.8 NA Plan-Apochromat objective) were collected for the GFP fluorescence channel and the transmitted light channel to locate cells of interest within the context of their neighboring cells, and to visualize the alphanumeric pattern on the grid for correlation with electron microscopy. Next, high resolution confocal stacks of cells of interest were acquired for GFP and MitoTracker Deep Red fluorescence channels and transmission light (126 x 126 × 541 nm^3^ voxels; 40x/1.3 NA Plan-Neofluar objective). Per dish, three spots were selected for further processing. Next, cells were post-fixed in EM fixative (4% paraformaldehyde, 2.5% glutaraldehyde in 0.1 M sodium cacodylate buffer) for 1 h at RT and washed 3 times with 0.1 M sodium cacodylate buffer, followed by fixation overnight at 4°C. After washing, cells were post-fixed in 1% osmium tetraoxide with potassium ferricyanide in 0.1 M sodium cacodylate buffer pH 7.2 at RT for 1 h. Afterward, samples were washed with ddH_2_O and subsequently dehydrated through a graded series of ethanol, including a bulk staining with 1% end concentration of uranyl acetate at the 50% ethanol step followed by embedding in Spurr’s resin. Ultrathin sections of a gold interference color were cut using an ultra-microtome (Leica EM UC6) followed by a post-staining in a Leica EM AC20 for 40 min. in uranyl acetate and for 10 min. in lead stain at 20°C. Sections were collected on formvar-coated copper slot grids and images were acquired with a JEM 1400plus transmission electron microscope (JEOL, Tokyo, Japan) operating at 80 kV. To find back HSPB8-GFP cells of interest at the electron microscope, we made use of the grid coordinates and of cell morphology, including information of the transmission light microscopy and the MitoTracker images. Overlay images of light microscopy (GFP, confocal) with transmission electron micrographs were generated with the Correlia plugin [81] of ImageJ [82].

### GFP expression time course experiment

HeLa cells were transfected with GFP-WT, GFP-mCTR or GFP-CE constructs as previously described. GFP expression was followed over time with the Incucyte® S3 Live-Cell Analysis System (Sartorius) by taking pictures every hour at fixed positions with a 20x objective. Raw uncalibrated 16-bit images were exported from the Incucyte software and analyzed in ImageJ [79,83].

### Co-immunoprecipitation

HEK293T-HSPB8-V5 were transfected with untagged pCi-HSPB8 constructs using Lipofectamine3000/P3000 reagent and following the manufacturers’ instruction (Invitrogen, L3000001). Forty-eight h after transfection, HEK293T-HSPB8-V5 cells were harvested and then centrifuged at 100 x g for 5 min at 4°C. Pellets were resuspended in RIPA (0.15 M NaCl [Sigma-Aldrich, S3014], 0.8% sodium deoxycholate [Sigma-Aldrich, D6750], 100 mM sodium orthovanadate [Sigma-Aldrich, S6508], 50 mM NaF [Sigma-Aldrich, S7920], 5 mM sodium iodoacetate [Sigma-Aldrich, I2512], 0.05 M Tris HCl, pH 7.7, 10 mM EDTA [Sigma-Aldrich, E6758], pH 8, 0.08% SDS [Sigma-Aldrich, L3771] and Triton X-100 [Sigma-Aldrich, X100]) buffer with protease inhibitors cocktail (Sigma-Aldrich, P8340) and then centrifuged at maximum speed. For the immunoprecipitation, SureBeads Protein A Magnetic Beads (Bio-Rad, 161–4013) were used, following the manufacturers’ instructions. An anti-BAG3 (Abcam, ab47124) and an anti-V5 (D3H8Q) (Cell Signaling Technology, 13202) antibodies were used for co-immunoprecipitation. Samples were then run on SDS-PAGE and western blot was performed (see western blot and FRA section).

### Measurement of number of aggregates per cell and aggregate size

For the different experimental conditions, images acquired with identical settings on a Zeiss LS700 laser scanning confocal microscope (Plan-Neofluar 40x/1.30 objective, 2048x2048, 0.078 µm pixels) were analyzed with the Fiji distribution of ImageJ [79]. Individual cells were delineated with the polygon selection tool and saved to the ROI manager. A macro script was employed to segment the bright HSPB8 aggregates in every cell by correcting for the background signal (subtraction of mean + 2x stdev of intensity), intensity thresholding (Triangle method) and particle size filtering (exclude particles <25 pixels), and subsequently extracting the number of aggregates per cell and their size (Analyze Particles command).

### Calculation of myoblast differentiation index, fusion index, and MYOG-positive nuclei

For the calculation of the differentiation and fusion index, differentiated myoblasts (day 7) were fixed and stained for MYH (myosin heavy chain) as described above. Per condition, 6 image z-stacks were acquired at random positions a Zeiss LS700 laser scanning confocal microscope (Plan-Apochromat 20x/0.8 objective, 512 × 512 pixels of 625 nm). For every stack, all nuclei were manually counted and categorized into three types: nuclei in MYH-negative cells (type 1), nuclei in MYH-positive cells containing only one nucleus (type 2), and nuclei in MYH-positive cells containing more than one nucleus (type 3). The differentiation index was calculated as the percentage of nuclei in MYH-positive cells (=$\frac{type\;\;2+3}{type\;\;1+2+3}$). The fusion index was calculated as the percentage of nuclei in fused myotubes (containing more than one nucleus) out of the total nuclei (=$\frac{type\;\;3}{type\;\;1+2+3}$). Data is representative of one differentiation experiment and per stack between 91 and 182 nuclei were evaluated, corresponding to a of total 680–872 nuclei per condition.

To measure the proportion of MYOG-positive nuclei, differentiated myoblasts (day 7) were fixed and stained for MYOG as described above. Per condition, image z-stacks were acquired at 8 random positions on a Nikon ECLIPSE Ti2 inverted microscope using a 20x PlanApo Λd 0.75NA objective. Maximum intensity projections of the z-stacks were created and used to measure intensities with the Fiji distribution of ImageJ [79]. To this end, nuclei were segmented in the Hoechst33342 nuclear channel using the ImageJ Stardist plugin [84], and in every nucleus mean intensities were measured in the MYOG fluorescence channel. After plotting the distribution of intensities for all nuclei, a fixed intensity threshold value was applied based on two evident populations to categorize every nucleus as either MYOG-positive or MYOG-negative (threshold value 700 in violin plot of Figure 7B). Data is representative of one differentiation experiment and per stack between 493 and 1206 nuclei were evaluated, corresponding to a total of 5918–8705 nuclei per condition.

### Statistics

One-Way ANOVA with Tukey’s multiple comparisons test, two-way ANOVA with Šídák’s multiple comparisons test or one-Way ANOVA with Dunnett’s multiple comparisons test were performed, as indicated in each figure, and using PRISM software (GraphPad Software).

### Molecular modeling

Models of N-Δ94 HSPB8_WT and fs mutants were built with knowledge-based homology modeling using HSPB1 as template (PDB: 6DV5). The four generated HSPB8 systems were submitted to 200 ns molecular dynamics in the NPT ensemble at 300 K. The most representative structure (medoid) of each system was extracted after clustering the trajectories using as metric the RMSD of the C-terminal loop. Aggregation propensity scores (AggScore) were then computed on these structures, as described [85]. All computations were carried out using the Schrödinger Suite 2022–2 (Schrödinger, LLC, New York, NY, 2021) with Desmond (Desmond Molecular Dynamics System, D. E. Shaw Research, New York, NY, 2021).

## Supplementary Material

Supplemental Material
