# Supplementary material for: HSPB8 frameshift mutant aggregates weaken chaperone-assisted selective autophagy in neuromyopathies
Source: Autophagy. 2023 Feb 28;19(8):2217–39. doi: 10.1080/15548627.2023.2179780 (PMC10351472; doi:10.1080/15548627.2023.2179780)
Supplement: Supplemental Material [file KAUP_A_2179780_SM9982.docx]

**Supplementary data**

**
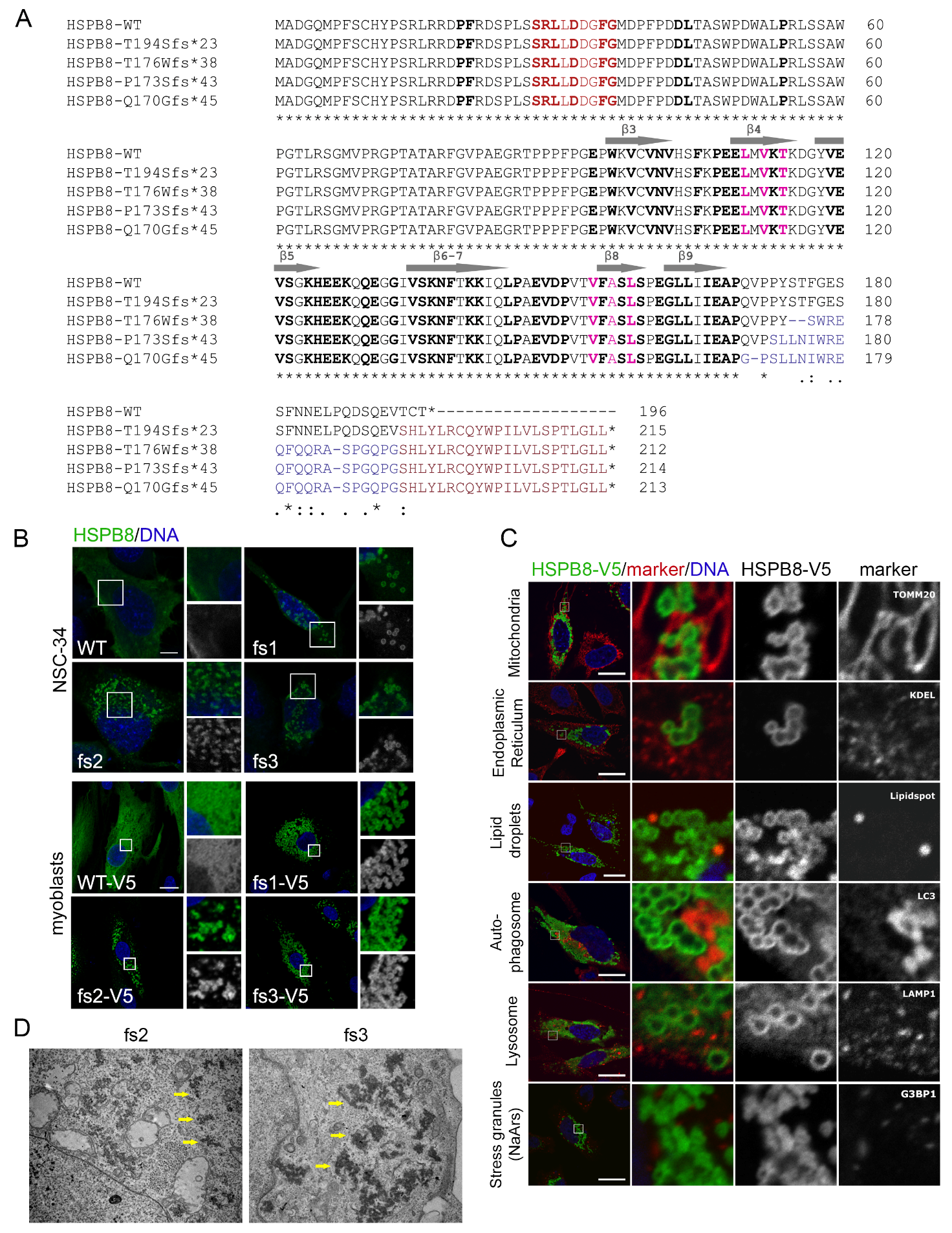
Figure S1.** The HSPB8_fs mutants sequences analyses reveal a common C-terminal extension causing their cytoplasmic aggregation. (**A**) Alignments of HSPB8_WT and HSPB8_fs mutant protein sequences (predicted) revealed a common mutated CTR (mCTR, blue) for p.P173Sfs*43 and p.Q170Gfs*45 mutants and a common C-terminal extension (CE, red) for all HSPB8_fs. Letters in bold and the arrows indicate the conserved amino acids among HSPBs and the β-sheets, respectively, based on the alignment previously reported [1]. The SRLFDQxFG motif is indicated in red, the motifs responsible for BAG3 binding are indicated in pink. (**B**) Top, immunofluorescence analysis of NSC34 cells transiently transfected with untagged HSPB8 constructs. HSPB8 (α-HSPB8) is in green, nuclei were stained with DAPI; scale bar: 10 μm. Bottom, immunofluorescence analysis of human myoblasts transduced with V5-tagged HSPB8 constructs. HSPB8 (α-V5) is in green, nuclei were stained with DAPI; scale bar: 20 μm. The constructs were abbreviated as follows: untagged HSPB8_WT (WT), HSPB8_fs mutants (fs1, fs2, fs3), V5-tagged HSPB8_WT and fs mutants (WT-V5, fs1-V5, fs1-V5, fs3-V5). (**C**) Immunofluorescence analyses of HeLa cells transiently transfected with a representative V5-tagged HSPB8_fs mutant (green) and stained for intracellular markers (red): TOMM20 (translocase of outer mitochondrial membrane 20) for mitochondria, KDEL sequence for endoplasmic reticulum, Lipid spot for lipid droplets, MAP1LC3B/LC3 for autophagosomes, LAMP1 (lysosomal associated membrane protein 1) for lysosomes, G3BP1 (G3BP stress granule assembly factor 1) upon sodium arsenite treatment for stress granules. Nuclei were stained with DAPI; scale bar: 20 μm. (**D**) Electron microscopy analysis on HeLa cells transiently transfected with HSPB8 mutants fs2 and fs3. Yellow arrows indicate protein dense aggregates (dark structures).


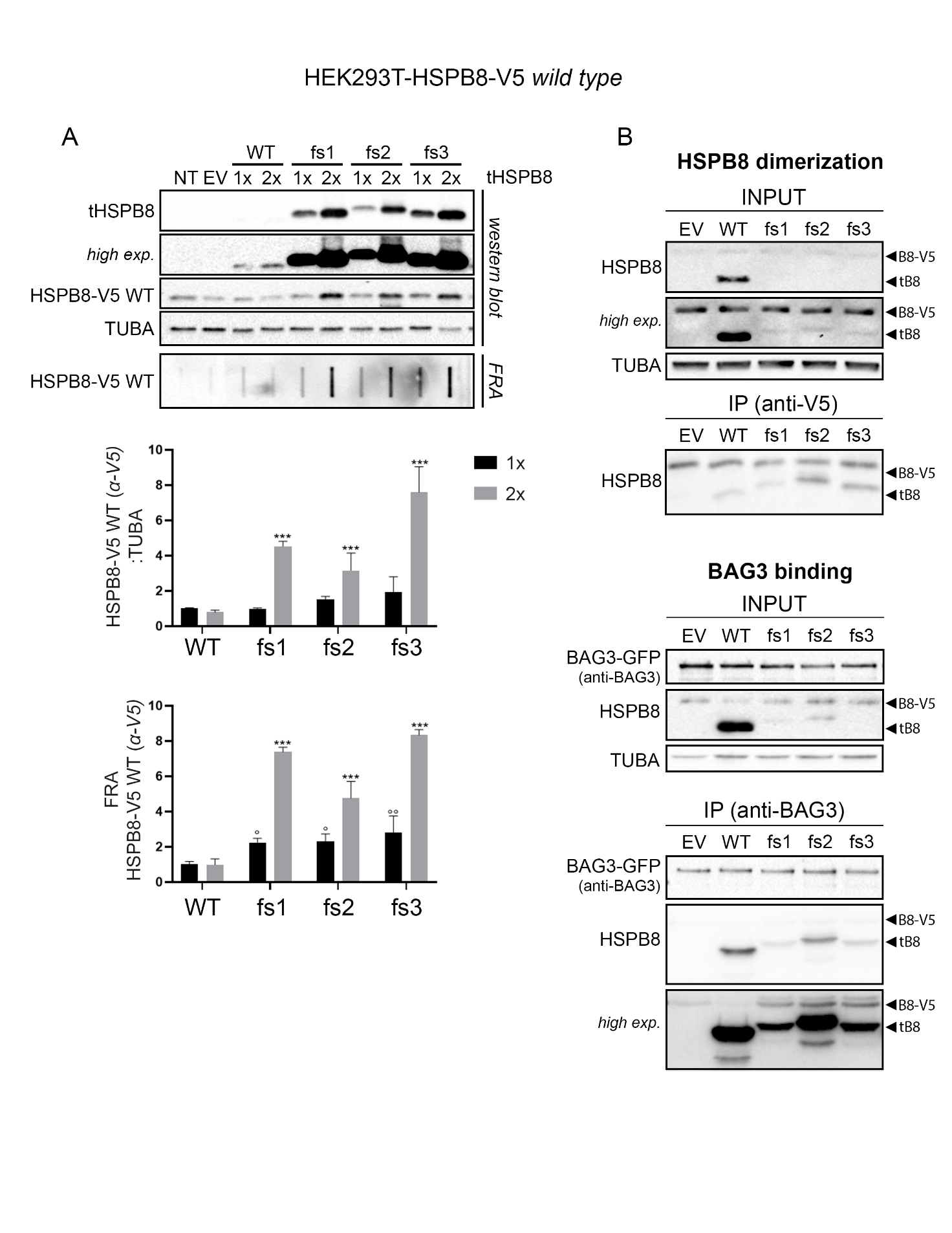
**Figure S2.** The HSPB8_fs mutants dimerize with HSPB8_WT decreasing its solubility and interact with BAG3. (**A**) Western blot and filter retardation assay of HEK293T cells stably expressing V5-tagged HSPB8_WT, not transfected (NT) or transiently transfected with increasing amounts of untagged HSPB8 constructs (tHSPB8, 0.5 μg [1x] or 1 μg [2x] plasmids) or an empty vector. Bar graphs report mean values (± SD) of densitometry of HSPB8-V5_WT, on TUBA/tubulin for western blot data (n=3). All graphs are normalized to untagged HSPB8_WT (1x). Two-way ANOVA with Dunnett’s test was performed: ° p<0.05, °° p<0.01, *** p<0.001. (**B**) Western blot analyses of RIPA-soluble protein lysates (INPUT) and co-immunoprecipitated samples (IP) from HEK293T cells stably overexpressing V5-tagged HSPB8_WT and transiently transfected with untagged HSPB8 constructs and GFP-tagged BAG3. HSPB8 homodimerization was evaluated by using an anti-V5 tag antibody; HSPB8 binding to BAG3 and competition with HSPB8-V5 was evaluated by using an anti-BAG3 antibody. B8-V5 indicates the V5-tagged HSPB8 WT, the tB8 indicates the transiently transfected untagged HSPB8s. The constructs were abbreviated as follows: empty vector (EV), HSPB8_WT (WT), HSPB8_fs mutants (fs1, fs2, fs3).

**
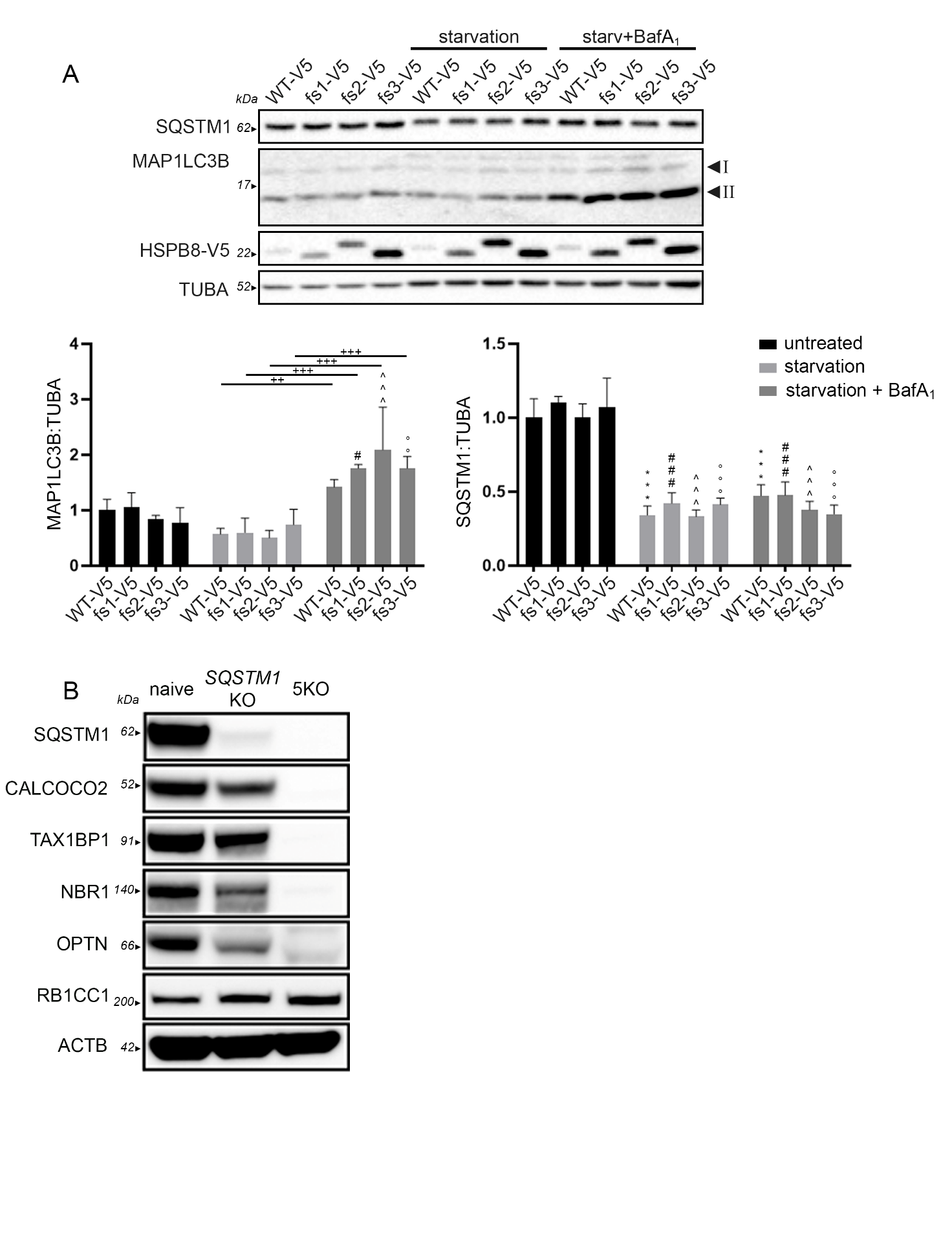
Figure S3.** Interplay between HSPB8_fs mutants and the autophagic pathway. (**A**) Western blot analysis on HeLa cells transiently transfected with V5-tagged HSPB8 constructs and analyzed for autophagy activity. LC3 and SQSTM1 were analyzed in basal (untreated) conditions, upon starvation (serum depletion, 24 h) with or without bafilomycin A_1_ (BafA_1_, 3 h, 10 nM). Bar graphs report mean values (± SD) of densitometry of MAP1LC3B and SQSTM1 on TUBA. All graphs are normalized to the untreated HSPB8_WT-V5. Two-way ANOVA with Tukey’s test was performed (n=3). Treatments versus untreated conditions: # p< 0.05; °° p<0.01; ***, ###, ^^^, °°° p<0.001. BafA1 treatment versus starvation: ++ p<0.01, +++ p<0.001. (**B**) Western blot analysis on HeLa parental cells (naive), HeLa *SQSTM1* KO or pentaKO (5KO) cell lines for autophagic receptors expression.

**
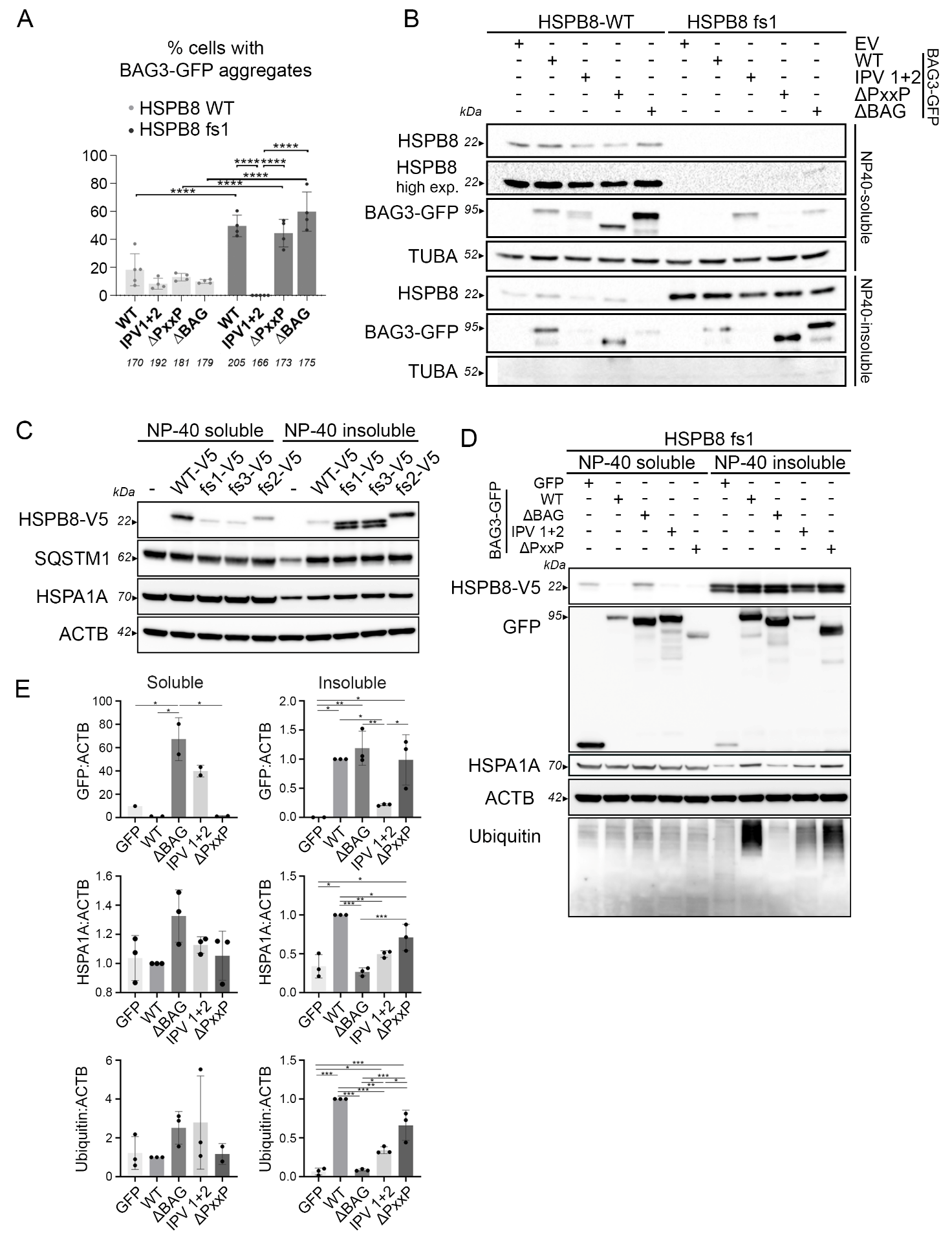
Figure S4.** Effect of HSPB8_fs on BAG3 and role of BAG3 on HSPB8_fs mutant aggregates formation. (**A**) Bar graph reports the percentage of NSC34 cells displaying BAG3-GFP aggregates from figure 5B. Cells were manually counted from four or five random fields. The mean number of cells per field was 42 and the number of counted cells is reported on the bottom of the graph. Two-way ANOVA with Šídák’s multiple comparisons test was performed: **** p<0.0001. (**B**) Western blot analyses after NP-40 soluble/insoluble extraction of NSC34 transiently co-transfected with an empty vector or BAG-GFP constructs and with the untagged HSPB8_WT or a representative HSPB8_fs mutant construct (fs1). (**C**) Western blot analysis of NP-40 soluble/insoluble extracts of HeLa *BAG3* KO cells lines transfected with V5-tagged HSPB8 constructs. (**D**) Western blot analyses after NP-40 soluble/insoluble protein extraction of HeLa *BAG3* KO cells transiently transfected with eGFPN1 or BAG-GFP constructs (WT or mutated) and with a V5-tagged HSPB8_fs mutant construct (fs1). (**E**) Bar graphs report mean values (± SD) of densitometry of GFP, HSPA1A, ubiquitinated proteins on ACTB/actin beta from panel S4D. All graphs are normalized to cells expressing BAG3-GFP-WT (WT). One-way ANOVA with Tukey’s test was performed: * p<0.05, ** p<0.01, *** p<0.001; n=3. The constructs were abbreviated as follows: empty vector (EV), V5-tagged HSPB8_WT (WT-V5) and HSPB8_fs mutants (fs1-V5, fs2-V5, fs3-V5), untagged HSPB8_WT (WT) and HSPB8_fs1 mutant (fs1), GFP-tagged BAG3s (BAG3-GFP).

**
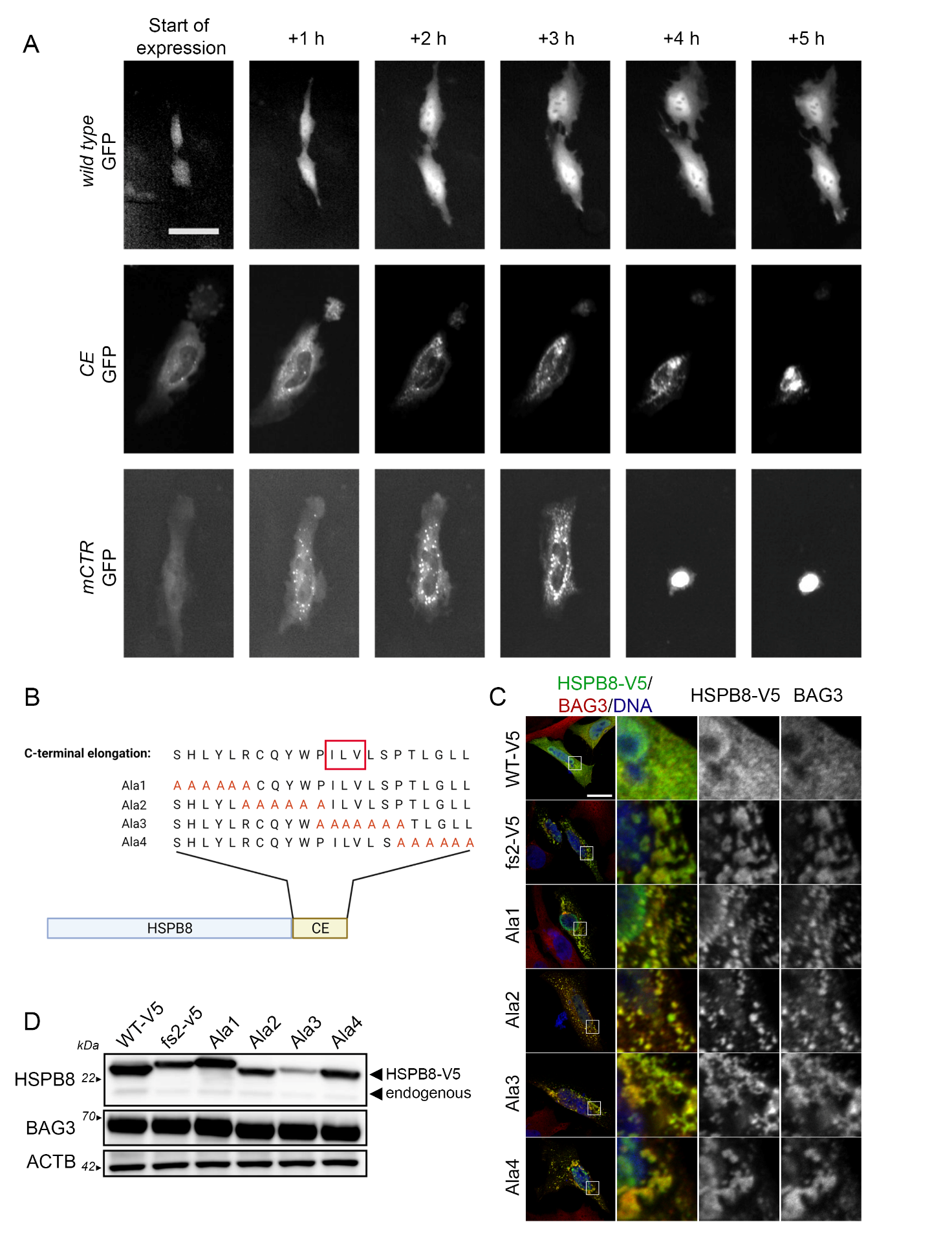
Figure S5.** The role of the mutated C terminus in protein aggregation. (**A**) Live-cell time-lapse imaging of HeLa cells transiently transfected with eGFP constructs (GFP-WT, GFP-CE or GFP-mCTR). Images were captured once per hour, from 9 to 13 h after transfection; scale bar: 100 μm. (**B**) Sequences of the HSPB8 constructs carrying the CE tract used for Ala-scanning (Ala 1-4). (**C**) Immunofluorescence analysis of HeLa transiently transfected with V5-tagged HSPB8 constructs (HSPB8_WT, HSPB8_fs2 or HSPB8 Ala1-4 mutants). Immunofluorescence was performed against HSPB8 (green) and BAG3 (red). Nuclei were stained with DAPI; scale bar: 20 μm. **(D)** Western blot results of HeLa transiently transfected with V5-tagged HSPB8 constructs (HSPB8_WT, HSPB8_fs2 or HSPB8 Ala1-4 mutants).

**References**

1. Boelens WC. Structural aspects of the human small heat shock proteins related to their functional activities. Cell Stress Chaperones [Internet] 2020; 25:581–91. Available from: https://www.ncbi.nlm.nih.gov/pubmed/32253739
